# Paleoceanographic Insights on Recent Oxygen Minimum Zone Expansion: Lessons for Modern Oceanography

**DOI:** 10.1371/journal.pone.0115246

**Published:** 2015-01-28

**Authors:** Sarah E. Moffitt, Russell A. Moffitt, Wilson Sauthoff, Catherine V. Davis, Kathryn Hewett, Tessa M. Hill

**Affiliations:** 1 Bodega Marine Laboratory, University of California Davis, Bodega Bay, California, United States of America; 2 Graduate Group in Ecology, University of California Davis, Davis, California, United States of America; 3 Marine Conservation Institute, Glen Ellen, California, United States of America; 4 Department of Earth & Planetary Sciences, University of California Davis, Davis, California, United States of America; 5 Department of Civil and Environmental Engineering, University of California Davis, Davis, California, United States of America; CAS, CHINA

## Abstract

Climate-driven Oxygen Minimum Zone (OMZ) expansions in the geologic record provide an opportunity to characterize the spatial and temporal scales of OMZ change. Here we investigate OMZ expansion through the global-scale warming event of the most recent deglaciation (18-11 ka), an event with clear relevance to understanding modern anthropogenic climate change. Deglacial marine sediment records were compiled to quantify the vertical extent, intensity, surface area and volume impingements of hypoxic waters upon continental margins. By integrating sediment records (183-2,309 meters below sea level; mbsl) containing one or more geochemical, sedimentary or microfossil oxygenation proxies integrated with analyses of eustatic sea level rise, we reconstruct the timing, depth and intensity of seafloor hypoxia. The maximum vertical OMZ extent during the deglaciation was variable by region: Subarctic Pacific (~600-2,900 mbsl), California Current (~330-1,500 mbsl), Mexico Margin (~330-830 mbsl), and the Humboldt Current and Equatorial Pacific (~110-3,100 mbsl). The timing of OMZ expansion is regionally coherent but not globally synchronous. Subarctic Pacific and California Current continental margins exhibit tight correlation to the oscillations of Northern Hemisphere deglacial events (Termination IA, Bølling-Allerød, Younger Dryas and Termination IB). Southern regions (Mexico Margin and the Equatorial Pacific and Humboldt Current) exhibit hypoxia expansion prior to Termination IA (~14.7 ka), and no regional oxygenation oscillations. Our analyses provide new evidence for the geographically and vertically extensive expansion of OMZs, and the extreme compression of upper-ocean oxygenated ecosystems during the geologically recent deglaciation.

## Introduction

**Figure 1:**
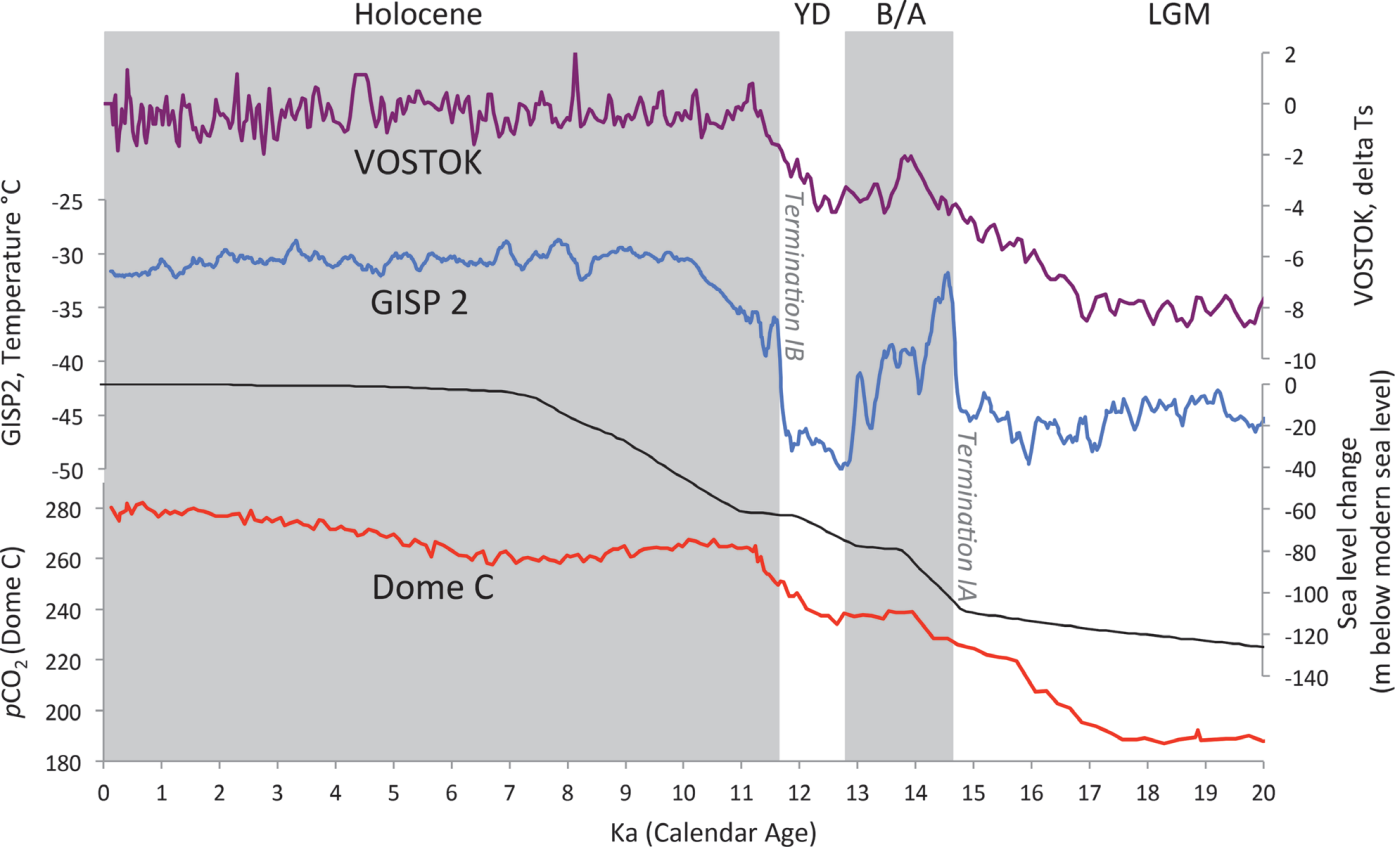
Deglacial changes in Antarctic temperature (Vostok ice core record, purple line) [146, 147], Greenland temperature (GISP2 ice core record, blue line) [106, 107], sea level (black line) [5, 6] and atmospheric pCO_2_ (red line) [3]. Glacial Termination IA (14.7 ka) is an event of rapid warming in the Northern Hemisphere, which initiates the warm interval of the Bølling-Allerød (B/A) from 14.7–12.9 ka. The Younger Dryas (YD), a reversal towards cool conditions from 12.9–11.7 ka, follows the B/A. The YD ends with glacial Termination IB (11.7 ka), a subsequent rapid warming event. Deglacial warming in the Southern Hemisphere begins at 18 ka.

Resolving records of global change through the most recent deglaciation event (18–11 ka) is one of the primary challenges to developing cohesive and robust theories regarding rapid climate change [1]. The last deglaciation was a profound event in the global climate system, wherein atmospheric [CO_2_] increased by 80–100 ppmv [2, 3], global average temperature rose 3–4°C [4], and sea levels rose ~110 m (Fig. 1) [5, 6]. This change was also accompanied by the pervasive loss of dissolved oxygen in the upper ocean [7], with unknown impacts on large marine ecosystems. The coupling between climate, carbon emissions and subsurface dissolved oxygen is a salient and critical element of anthropogenic climate change. The global inventory of ocean oxygen is predicted to decline between 1 and 7% by the year 2100, through stratification, ventilation reduction and decreased O_2_ solubility (e.g., [8]), and the hypoxic water volume in the global ocean is predicted to increase by 50% [9]. Climate model results beyond the 100-year window reveal extensive oceanic deoxygenation, on thousand-year timescales, under “business-as-usual” carbon emission scenarios, and show that oceanic deoxygenation is a fundamental and long-lasting property of anthropogenic carbon perturbation (e.g. [10]).

Hypoxia substantially degrades ecosystems through mass mortality events, the alteration of food-web structures and the loss of habitat (e.g., [11]). Changes in [O_2_] in the ocean interior have broad consequence for global biodiversity, marine economic resources and ocean management [12]. To grasp the scale of future hypoxia disturbance, the paleoceanographic (pre-instrumental) record of natural variability provides a critical analytical and interpretive window. Ocean sediments are climatic and environmental archives, which preserve geochemical, microfaunal and sedimentary evidence that record globally relevant Earth system events, similar to other climate records such as the Greenland Ice Sheet Project [13]. Recent investigations reveal that paleoceanographic investigations hold valuable insight into modern environmental conservation and management [14, 15].

Here we synthesize published continental margin sediment core records to investigate Oxygen Minimum Zone (OMZ) changes through the last deglaciation. We build on previous syntheses of oxygenation proxy records (e.g., [7, 16, 17]), and provide a focus on regional-scale sensitivity. By integrating sediment records, sea level change, and high-resolution bathymetry, we provide geospatially analyzed paleoceanographic data that are interpretive baselines for modern oceanography and global environmental change.

### The role and importance of OMZs

**Figure 2:**
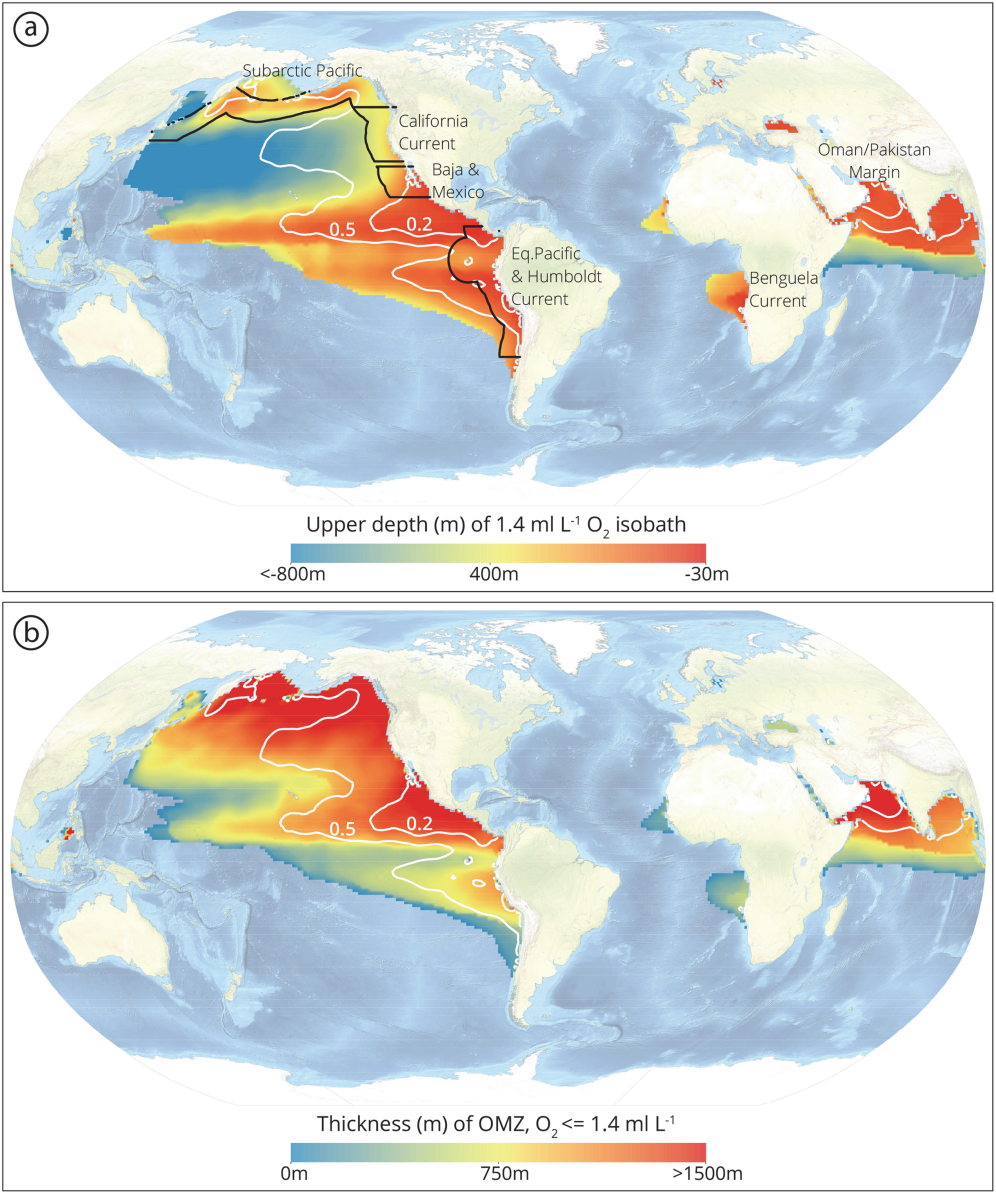
Global Oxygen Minimum Zones, including (a) Upper depth (in meters) of intermediate water hypoxia ([O_2_]<1.4 ml L^-1^) and (b) thickness (in meters) of intermediate water hypoxia ([O_2_]<1.4 ml L^-1^). The geospatial distributions of severely hypoxic [O_2_] minimums (of [O_2_] = 0.5 ml L^-1^ and [O_2_] = 0.2 ml L^-1^) are depicted on both panels as white lines. For the upper panel, regional blocks are defined by black lines to highlight where paleoxygenation reconstructions were completed. Data from World Ocean Atlas [192].

OMZs are tightly coupled to upwelling systems and Eastern Boundary Currents, such as the California Current, the Humboldt Current and the Benguela Current, as well as the Oman and Pakistan Margin in the Indian Ocean (Fig. 2). In these regions, respiration within the pycnocline depletes dissolved oxygen and simultaneously enriches seawater in the carbon and nitrogen byproducts of respiration [18]. Marine denitrification occurs within OMZs (e.g., [19, 20]) therefore the physical extent and intensity of OMZs is inherently coupled to the oceanic nitrogen cycle. OMZs form at shelf and upper slope depths, and are considered to be unique biological, geochemical and evolutionary environments, analogous to cold seep or deep-sea vent environments [21]. As continental margin ecosystems transition from well oxygenated surface waters to the hypoxic core of the OMZ ([O_2_] = 0.5–0.1 ml L^-1^), faunal diversity, trophic structures and physiological strategies change (e.g., [22, 23]). OMZ oxygenation gradients produce successional biological zonation and are fundamental habitat barriers for benthic and pelagic organisms [21].

For this work, we follow the hypoxia thresholds and categories defined in [24], which synthesizes the existing hypoxia vernacular, to draw thresholds that are biologically meaningful (Table 1). *Mild hypoxia* begins at [O_2_]<2.45 ml L^-1^ and is the threshold where sensitive species exhibit avoidance reactions. *Intermediate hypoxia*, often referred to as “coastal hypoxia”, occurs at [O_2_]<1.4 ml L^-1^ and is the threshold wherein ecosystems are dominated by organisms with adaptive features. *Severe hypoxia* ([O_2_]<0.5 ml L^-1^) is a threshold at which mass mortality is induced for most organisms, past which only highly specialized species can survive [24].

**Table 1:** Hypoxia categories and associated oxygen concentrations, partial pressures and saturations for each category.

**Hypoxia Category**	**ml O_2_ L^-1^**	**µmol O_2_ kg^-1^**	**mg O_2_ L^-1^**	***p*O_2_**			**% O_2_saturation**		
				*T = 25°C*	*T = 17°C*	*T = 12°C*	*T = 25°C*	*T = 17°C*	*T = 12°C*
Mild	2.45	107	3.5	106	93	84	51≈50	45	40
Intermediate	1.4	61	2.0	60	53	48	29≈30	25	23
Severe	0.5	22	0.71	22	19	17	11≈10	9	8

Salinity (34 psu) and hydrostatic pressure (P = 10 bar) are assumed constant. Temperature columns indicate the temperature used for partial pressure and saturation calculations at the associated concentrations. Data table adapted from *Hofmann et al.*, [24]. The intermediate category is described as “coastal” in Hofmann et al., [24]. We refrain from using this term here, to prevent confusion between hypoxia categories and offshore habitat locations

### Oxygenation proxies in paleoceanographic records

**Figure 3:**
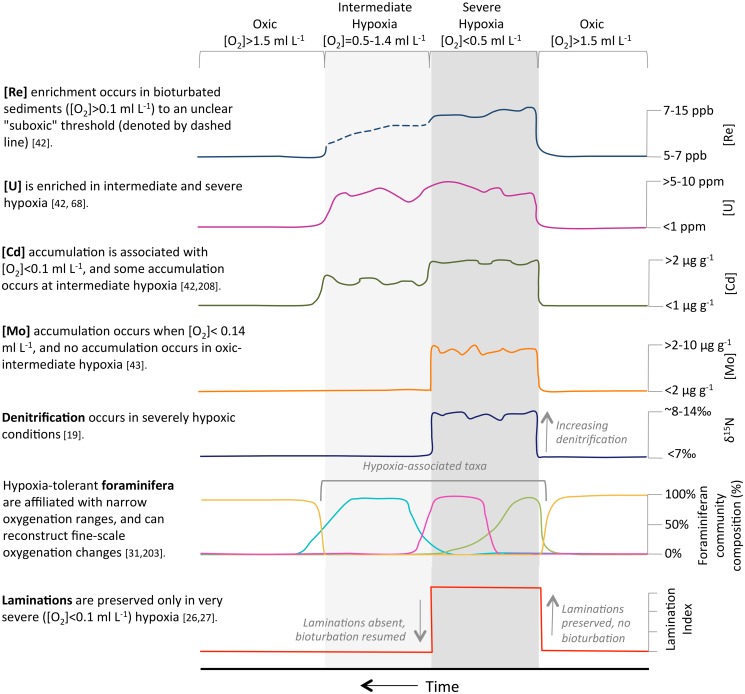
Schemata of a multi-proxy approach to interpreting hypoxia categories, including severe hypoxia ([O_2_]<0.5 ml L^-1^), intermediate hypoxia ([O_2_]>0.5–1.5 ml L^-1^) and mild hypoxia to oxic conditions ([O_2_]>1.5 ml L^-1^). These hypoxia categories are detailed in Table 1, and follow *Hofmann et al.*, [24]. Hypoxia proxies include [Re], [Mn], [U], [Cd], [Mo], δ^15^N, foraminiferan communities, and sedimentary laminations. Units for each proxy reflect the cited literature, which constrains the proxy to a specific oxygenation category.

We employ a multi-proxy approach to oxygenation reconstructions here, and include core data across sedimentary, faunal and geochemical proxies (Table 1). Fig. 3 depicts, in schemata form, how regional multi-proxy oxygenation data are interpreted, and we discuss each proxy below.

**Sedimentary structures**. Severe hypoxic conditions preclude benthic macrofauna, preventing bioturbation and thereby allowing for the preservation of laminated sediments (Table 2) [25–27]. For example, annual sediment laminations (i.e. unmixed, fine-grained sediment displaying distinct, continuous layering) are formed in modern California margin basinal features at [O_2_]<0.1 ml L^-1^ [25, 28]. Laminations are formed under the absence of bioturbating invertebrates [29] and sufficiently high organic carbon export from the surface [16], and are one of the clearest indicators of severe hypoxia in benthic environments.

**Table 2:** Seafloor hypoxia proxies for paleoceanographic reconstructions, partitioned by the thresholds and capacity each proxy has to record fine-scale changes in seafloor hypoxia, as well as organic flux to the seafloor [24].

**Proxy**	**Mild Hypoxia ([O_2_] = 2.45–1.4 ml L^-1^)**	**Intermediate Hypoxia ([O_2_] = 0.5–1.4 ml L^-1^)**	**Severe Hypoxia ([O_2_]<0.5 ml L^-1^)**	**Indicator of increased organic flux to seafloor?**	**Notes**
**Laminations**	Not Present		Laminations only occur with extremely severe seafloor hypoxia ([O_2_]<0.1 ml L^-1^), where bioturbating benthic fauna are not present [26, 27].	Yes. Lamination formation requires high surface export [16].	
**Foraminifera**	Above 2 ml L^-1^, foraminiferan composition is likely not altered by changes in bottom water oxygenation [199].	Foraminiferan communities in intermediate-severely hypoxic sediments are dense and associated with opportunistic taxa and specific morphologies [200–202]. Marker species, affiliated with a narrow oxygenation range, can be used to reconstruct seafloor oxygenation on a very fine-scale [31, 203].		Organic flux to the seafloor alters the composition and density of foraminiferan communities [204, 205], however interpretation of these community traits is not straightforward in a low oxygen setting (e.g., [206]).	Foraminifera are well adapted to the extreme chemical heterogeneity of oxygenation, methane enrichment, organic flux and sulfur-reducing environments found on continental margins [207].
**δ^15^N**	No significant denitrification, with no isotopic fractionation.		Denitrification occurs in severely hypoxic conditions ([O_2_]<0.23 ml L^-1^) [19]. The isotopic signal of denitrification reflects regional changes in biologically available N pool [35].	δ^15^N is an indirect record of organic flux, as photosynthesis isotopically fractures the nitrogen pool.	The δ^15^N of particulate nitrogen varies with the degree of surface nutrient utilization, commonly termed productivity [37, 38], and water column denitrification [128]. For continental margins, sediment denitrification contributes to the isotopic signal [19, 20]. Denitrifying bacteria are facultative anaerobes, meaning they are able to respire either oxygen or nitrogen oxides.
**Molybdenum [Mo]**	No accumulation increase [43].		Accumulation occurs when [O_2_]<0.45 ml L^-1^ [43]. High accumulation rates (>2 μg g-^1^) are related to the presence of anoxia [36].	Yes, non-lithogenic particulate Mo, associated with sinking particles, contributes ≤15% of authigenic Mo accumulation [43].	Mo is a biologically-essential, and is conservative in behavior. The pathway for authigenic Mo accumulation in sediment is unresolved, however it is clear that sulfide concentrations must be >2 μM for active Mo formation [43] Concurrent enrichment of Mo and Re is an indicator of an anoxic depositional environment [41].
**Cadmium, [Cd]**	No accumulation increase	Some accumulation at lower levels in “coastal hypoxia” [208].	Significant Cd enrichment (>2 ppm) is associated with laminated depositional records, a suboxic to anoxic seafloor and the presence of trace presence of H_2_S [42].	Composition of Cd in sinking particles is similar to plankton material; therefore changes in productivity may affect levels of enrichment [41].	Cd is involved with nutrient cycling and scavenging. It is released in sediment during the diagenesis of organic matter, forming insoluble sulfides when trace amounts of H_2_S are present [209]. Cd enrichment occurs in surface sediments [41].
**Uranium, [U]**	No accumulation increase	U is enriched in “suboxic” and anoxic sediments as a result of reduction and precipitation (>5μg g^-1^) [210].	U enrichment occurs in bioturbated sediments (>0.1 ml L^-1^), indicating enrichments at an unclear “suboxic” threshold [42].	Unclear, as the composition of U in plankton is not known.	U is conservative in behavior, and the accumulation of U may be kinetic in nature. U accumulation may be promoted by kinetic effect, in areas of low sedimentation rates [17, 211, 212]. U enrichment occurs in subsurface sediments [41].
**Rhenium, [Re]**	No enrichment	Re enrichment occurs in bioturbated sediments ([O_2_]>0.1 ml L^-1^), indicating enrichment at an unclear “suboxic” threshold [42].	Re is highly enriched in “suboxic” and anoxic sediments [42, 210].	Unclear, as the composition of Re in plankton is not known.	Re is conservative in behavior, diffuses across the sediment-water interface, and the direct mechanism for accumulation is unknown. Free H_2_S is not critical for the accumulation of Re [210]. Concurrent enrichment of Mo and Re is an indicator of an anoxic depositional environment [41].

A diagram for how multiproxy records are interpreted into paleoxygenation categories is shown in Fig. 3.

**Microfossils**. Oxygen is a physiochemical parameter that acts as a limiting factor, thereby determining the distribution of where organisms can live based on their physiological requirements. Within the optimal range of a limiting factor, a species will reach maximum competitiveness and maximum abundance [30], resulting in tight faunal affiliations with specific oxygenation ranges. Benthic foraminifera have species-specific oxygenation thresholds and therefore marker taxa function as oxygenation proxies [31]. Metrics of foraminiferan community structure, such as diversity and density, also record changes in seafloor oxygenation [22, 23, 32]. Due to their opportunistic responsiveness to environmental change, Foraminifera are ideally suited for high-resolution oxygenation reconstructions [32–34].

**Geochemistry**. Isotopic and trace element geochemical proxies of oxygenation are complex, as the cycling, preservation and attribution of these elements can be controlled by processes such as of surface productivity and flux, water column processes, sediment-water interface flux and diffusion, and sub-surface sedimentary processes. Denitrification in continental margin regions occurs under hypoxic conditions in both the water column and sediment ([O_2_]<0.23 ml L^-1^) [19, 20], and changes in the nitrogen isotopic signal are a product of regional changes in the biological available N pool [35]. Although intense denitrification is often indicative of severe hypoxia, more subtle changes in δ^15^N can be attributed to a range of related localized factors, such as source water and nutrient availability [19, 36–39]. Redox sensitive trace elements such as cadmium (Cd), uranium (U), chromium (Cr) and rhenium (Re) occur in bulk sediment and carbonate fossils, and can be used to characterize the redox state of the seafloor as well as functioning as proxies of paleoenvironmental redox changes [40–43]. Paleoproductivity proxies are useful supportive proxies in OMZ reconstructions, due to the mechanistic coupling between surface export and subsurface respiration [44]. Additionally, the δ^13^C record of planktonic carbonate reflects the surface ocean isotopic pool, and can be used to reconstruct surface water productivity and carbon export [45–47].

## Methods

### Sedimentary archives

We include cores in this review that met criteria for further geospatial analysis (Table 3). These criteria dictated sediment records that:

- exist in a geographic region that includes a modern OMZ,
- range in age from approximately 20–0 ka,
- have well-constrained chronologies (radiocarbon and/or isotope stratigraphy),
- include one or more primary proxies for hypoxia, as described above.

**Table 3:** Deglacial sediment core metadata and references for sites used in geospatial analyses.

**Core ID**	**Modern water depth (m)**	**Latitude**	**Longitude**	**Sedimentation rate (cm kyr^-1^) or Mass Accumulation Rate (MAR; g cm^-3^ kyr^-1^)**	^14^ **C dating**	*δ* ^18^ **O stratigraphy**	**Laminations**	**Microfossils**	**Geochemistry [δ^15^N]**	**Geochemistry [Mo]**	**Geochemistry [Cd]**	**Geochemistry [U]**	**Geochemistry [Re]**	**References**
MV0811–15JC	418	34° 22.2′ N	120° 7.8′ W	42.2–100	x	x		x						[103]
ODP 893A	576.5	34° 17.251′ N	120° 2.2′ W	140	x	x	x	x	x	x	x	x	x	[27, 29, 83, 85, 86, 88, 90, 100, 213]
MD02–2503	570	34° 16.48′ N	120° 2.24′ W	100		x	x	x						[89, 102, 103]
MD02–2504	481	34° 13.998′ N	119° 52.122′ W	80–100		x	x	x						[89, 102, 103]
ODP 1017E	955	34° 32.1′ N	121° 6.43′ W	23	x	x		x	x	x	x	x	x	[17, 94, 214]
F2–92–P3	786	35° 37.39′ N	121° 36.28′ W	~17	x		x			x				[40, 95, 96, 105]
F2–92–P34	610	35° 1.85′ N	121° 13.54′ W	~10	x		x			x				[96, 105]
F2–92–P40	760	35° 25.09′ N	121° 24.95′ W	~42	x		x			x				[40, 96, 105]
ODP 1019E	980	41° 40.9′ N	124° 55.8′ W	41	x	x	x		x	x	x	x	x	[42, 105, 215, 216]
F–8–90–G21	1650	37° 13.4′ N	123° 14.6′ W	4–23	x	x		x			x			[95–97]
Core JT96–09	920	48° 54.067′ N	126° 53.034′ W	5–169	x			x		x	x	x	x	[92, 217]
EW0408–85JC	682	59° 33.32′ N	144° 9.21′ W	9–500	x		x	x	x	x	x	x		[58, 67, 72, 74]
EW0408–11JC	183	55° 37.6′ N	133° 30.69′ W	37–280	x					x		x		[72]
CH84–14	978	41° 44′ N	142° 33′ E	55–208	x		x	x		x		x		[68, 179]
RAMA 44PC	2980	53° 0′ N	164° 39′ E	4–29	x			x				x		[68, 70, 71, 74]
GGC-55/JPC-56	818	27° 28.16′ N	112° 6.26′ W	83	x	x	x	x	x					[16, 128, 182, 218]
MV99-PC08	705	23° 28.2′ N	111° 36′ W	30	x		x		x	x	x			[16, 105, 121, 128, 182, 218–220]
MV99-PC14	542	25° 12′ N	112° 43.2′ W	108	x		x							[16, 105, 121, 219, 220]
NH15P	420	22° 4′ N	106° 28.8′ W	4–17	x	x	x	x	x	x	x	x	x	[16, 119, 120, 122, 219, 221, 222]
NH8P	1018	22° 23.3′ N	107° 4.5′ W	11.5–16	x	x	x	x	x					[16, 119, 120, 122, 126, 127, 221, 222]
MD02–2508	606	23° 27.91′ N	111° 35.74′ W	15–43	x		x		x		x			[16, 17, 120, 126, 127, 129, 221]
DSDP Site 480	655	27° 54′ N	111° 39′ W	77–114	x		x	x						[16, 17, 26, 129]
ODP Site 1242	1364	7° 51.352′ N	83° 36.418′ W	~10	x	x			x					[16, 26, 124, 181, 223]
W7706–37K	370	-13° 38′ S	76° 51′ W	~100	x		x		x					[124, 145, 151, 181, 223, 224]
W7706–40K	186	-11° 15′ S	77° 58′ W	*~55*	x		x		x					[145, 151, 224–226]
W7706–41K	410	-11° 21′ S	78° 7′ W	*N/A*	x				x					[145, 151, 224–226]
ME0005A-27JC	2203	1° 51.2′ N	82° 47.2′ W	~3 MAR	x	x			x			x		[149, 151, 227, 228]
ME0005A-24JC	2941	0° 1.2′ N	86° 27.6′ W	~4.5–12 MAR	x				x			x		[124, 149, 181, 227–229]
Y69–71P	2740	0° 6′ N	86° 28.8′ W	~3–6 MAR	x	x			x			x		[124, 149, 181, 229, 230]
P7	3085	2° 36′ N	83° 59.4′ W	~1.5–2.5 MAR	x				x			x		[148, 149, 230, 231]
TR163–31P	3209	-3° 36′ S	83° 57′ W	~3–9 MAR	x	x			x			x		[148, 149, 227, 228, 231–235]
TR163–19P	2348	2° 15.6′ N	90° 57′ W	~2–4 MAR		x			x			x		[149, 227, 228, 232–235]
GeoB7139–2	3269	-30° 12′ S	71° 59′ W	~13	x				x					[123, 149, 150, 181, 228, 232, 235, 236]
V19–30	3091	-3° 22.8′ S	83° 30′ W	~7		x			x			x		[123, 144, 150, 181, 236, 237]
RC13–140	2246	2° 52.2′ N	87° 45′ W	>5	x	x	x		x			x		[144, 237, 238]
RC11–238	2573	-1° 31.2′ S	85° 49.2′ W	>5	x	x	x		x			x		[144, 238]

Associated data provided here include modern water depth of core extraction (m), latitude and longitude, sedimentation (cm kyr^-1^) or mass accumulation rate (g cm^-2^ kyr^-1^), the presence of ^14^C dating or *δ* ^18^O stratigraphy, and the presence of published hypoxia proxy records (including laminations, microfossils, or geochemistry).

We rely on published chronologies for the deglacial and post-glacial core material, and do not reinterpret any chronologies. Regional archives are assembled on a unifying age axis, which is determined only by previously published chronologies. Sedimentation rates for sites <1000 mbsl span a minimum of 9 cm kyr^-1^ to a maximum of 500 cm kyr^-1^, with the majority sedimentation rate ranging from 20–100 cm kyr^-1^. Sedimentation rates for sites >1000 mbsl span a minimum of 4 cm kyr^-1^ to a maximum of 16 cm kyr^-1^. Mass Accumulation Rates (MARs) are published for deep sites (>2200 mbsl) and they range from 2–9 g cm^-3^ kyr^-1^.

Paleoxygenation was assessed based on proxy evidence available for each for core. If proxy evidence indicated hypoxia, we partitioned that signal into intermediate or severe hypoxia groups, based on the biologically-relevant classification scheme identified by *Hofmann et al.*, [24]. To ensure hypoxia designations are as conservative as possible, we refrained from any hypoxic designation without evidence. In practice, this meant we only designated hypoxia where the proxy evidence was explicit. In the absence of that evidence, we did not designate any hypoxia (i.e., in the absence of laminations, we did not designate a potential range from oxic-intermediately hypoxic). Importantly, we found no instance where multiple proxies within one archive produced conflicting hypoxia reconstructions.

Sedimentary archives were then assigned paleodepths based on the modern water depth for the core site, the age model of that core, and deglacial eustatic sea level change. Paleodepths were calculated using estimated global eustatic sea level fluctuations constructed from multiple deglacial sea level records (Fig. 1) [5, 6]. We acknowledge eustatic sea level is a simplification of local sea level trends through the deglaciation, which are also impacted by glacio-hydro-isostatic effects [48, 49].

### Bathymetric and Geospatial Analyses

Data visualizations applied to bathymetry, or submarine topography, provide unique perspectives into the distribution of seafloor ecosystems. Here we apply bathymetric masks associated with reconstructed paleoxygenation, wherein mask depths were chosen based on the extent of regional hypoxia across two or more cores at key temporal deglacial intervals. If only a single hypoxia record was available for a deglacial event, a hypoxic mask ±50 m from the core depth was applied. Oxic and hypoxic benthic surface area (km^2^) and water volume (km^3^) were quantified for temporal intervals through the deglaciation. To produce geospatial maps of paleoxygenation, we synthesized regional scale patterns in oxygenation, took into account eustatic sea level change, and analyzed the deglacial changes in both hypoxic seafloor surface area and hypoxic water volume from the SRTM30_PLUS global bathymetry dataset [50] using ArcGIS geospatial software [51]. Analyses were limited to the continental margin within a 400 nautical mile buffer offshore of the continental coastline. One exception to this is the inclusion of the seafloor within 400 nautical miles around the Galapagos Islands, in order to capture the associated cluster of deep core sites nearby. Within each region, the analysis was limited to the seafloor and water column above a specified isobath, selected below the deepest regional core depth.

**Figure 4:**
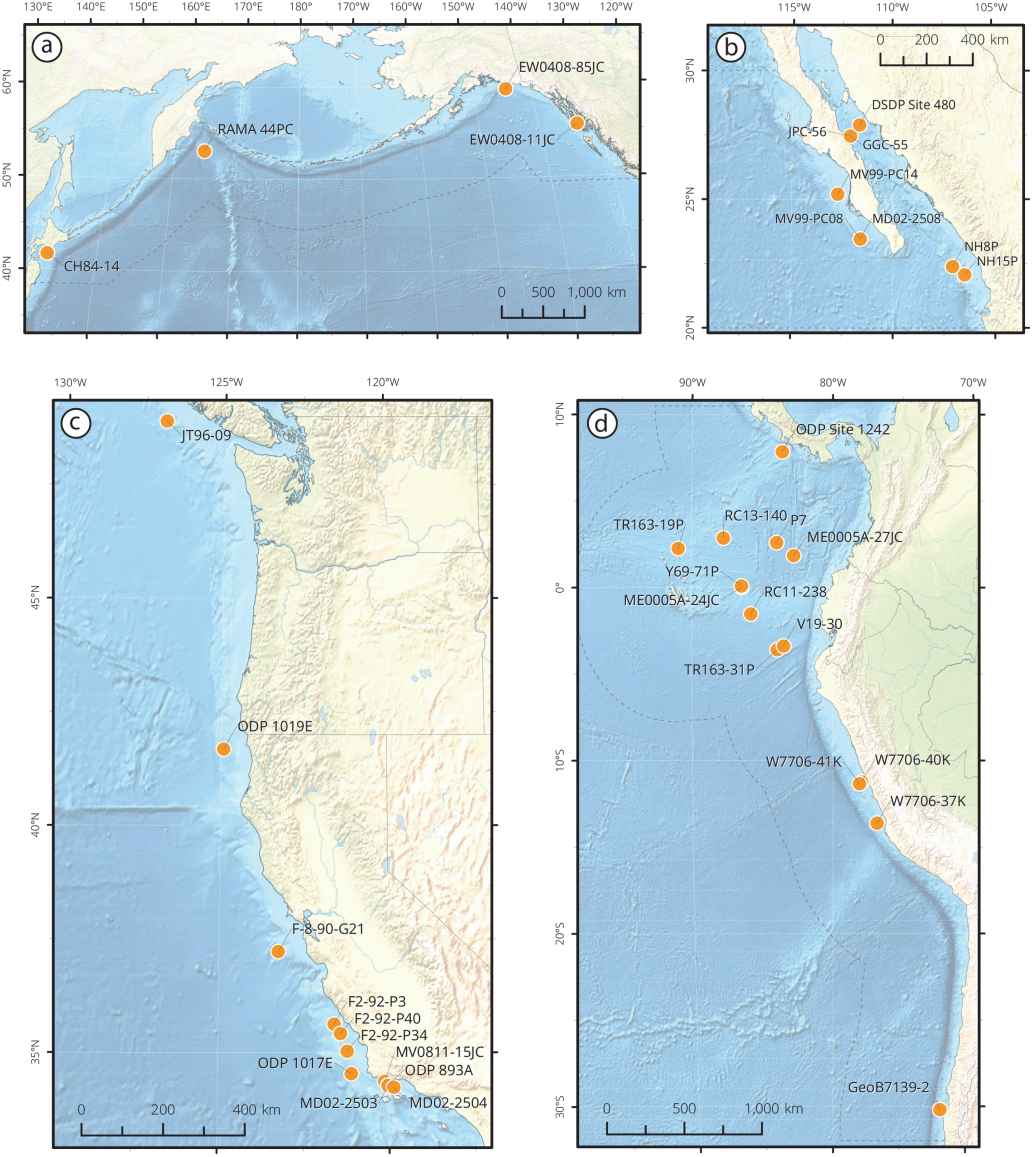
Deglacial sediment core locations for four Eastern Pacific regions, including (a) the Subarctic Pacific, (b) the Mexico Margin, (c) the California Current, and (d) the Equatorial Pacific and Humboldt Current.

We include four regions for which geospatial analyses were conducted: the Subarctic Pacific (SP; 0–3,200 mbsl; southern latitude limit at 38° 30′ N in the Western Pacific and 49° 30′ N in the Eastern Pacific), the California Current (CC; 0–2,400 mbsl; 31° 40′-49° 30′ N), Mexico Margin (MM; 0–1,200 mbsl; 20°-30° N) and the Humboldt Current and Equatorial Pacific (HC; 0–3,300 mbsl; 10° 30′ N-32° S) (Fig. 4). The Benguela Current (BC) and the Oman and Pakistan Margin (OPM) are discussed, however sediment records from these regions did not meet the criteria established to warrant geospatial analysis. We limit our statements of specific volume and surface area changes to Tables 4 and 5. These analyses represent the state of paleoceanographic records of deglacial OMZ expansion, including caveats associated with proxy records and limitations of the spatial resolution of core sites; our work may also be used to highlight existing knowledge gaps.

**Table 4:** Surface area calculations of deglacial oxygenation changes for four Eastern Pacific continental margins: Subarctic Pacific (SP), California Current (CC), Mexico Margin (MM), and the Humboldt Current and Equatorial Pacific (HC).

**Region**	**Ka (Calendar Age)**	**Regional margin surface area (km^2^), corrected for sea level rise**	**Total vertical extent of hypoxia (in meters)**	**Oxic upper ocean, from surface to upper subsurface hypoxic boundary (km^2^)**	**Total Oxic (km^2^)**	**Oxic (%)**	**Severe Hypoxia (km^2^)**	**Severe Hypoxia (%)**	**Intermediate Hypoxia (km^2^)**	**Intermediate Hypoxia (%)**
**SP**	14	1,177,000	2,298	426,000	603,000	51	574,000	49	0	0
	10	1,259,000	0	*N/A*	1,259,000	100	0	0	0	0
	4	1,325,000	100	335,000	1,167,000	88	158,000	12	0	0
**CC**	18	274,000	0	*N/A*	274,000	100	0	0	0	0
	14	285,000	1,232	60,000	153,000	54	50,000	17	83,000	29
	12	292,000	0	*N/A*	292,000	100	0	0	0	0
	10	297,000	1,232	80,000	165,000	56	10,000	3	122,000	41
	4	309,000	528	99,000	254,000	82	10,000	3	45,000	15
**MM**	18	158,000	100	109,000	148,000	94	10,000	6	0	0
	14	181,000	603	79,000	120,000	66	61,000	34	0	0
	10	216,000	518	111,000	154,000	71	61,000	28	0	0
	4	264,000	398	151,000	221,000	84	43,000	16	0	0
**HC**	18	2,648,000	100	716,000	1,370,000	52	0	0	1,278,000	48
	13	2,572,000	3,022	98,000	621,000	24	54,000	2	1,897,000	74
	10	2,507,000	2,749	185,000	421,000	17	18,000	1	2,068,000	82
	4	2,428,000	100	428,000	2,388,000	98	0	0	40,000	2

**Table 5:** Volumetric calculations of deglacial oxygenation changes for four Eastern Pacific continental margins: Subarctic Pacific (SP), California Current (CC), Mexico Margin (MM), and the Humboldt Current and Equatorial Pacific (HC).

**Region**	**Ka (Calendar Age)**	**Depth of seafloor analyzed (m), corrected for sea level rise**	**Regional margin volume (km^3^), corrected for sea level rise**	**Oxic upper ocean, from surface to upper subsurface hypoxic boundary (km^3^)**	**Total Oxic (km^3^)**	**Percent of margin oxic (%)**	**Severe hypoxia (km^3^)**	**Percent of margin severely hypoxic (%)**	**Intermediate hypoxia (km^3^)**	**Percent of margin intermediately hypoxic (%)**
**SP**	14	3,214	1,524,000	456,000	479,000	32%	1,045,000	68%	0	0%
	10	3,255	1,500,000	*N/A*	1,500,000	100%	0	0%	0	0%
	4	3,299	1,480,000	144,000	1,397,000	94%	83,000	6%	0	0%
**CC**	18	2,280	316,000	*N/A*	316,000	100%	0	0.00%	0	0%
	14	2,314	302,000	75,000	119,000	39%	86,000	28%	98,000	32%
	12	2,336	295,000	*N/A*	295,000	100%	0	0%	0	0%
	10	2,355	288,000	83,000	118,000	41%	18,000	6%	152,000	53%
	4	2,399	277,000	91,000	189,000	68%	17,000	6%	70,000	25%
**MM**	18	1,080	65,000	60,000	62,000	96%	2,000	4%	0	0%
	14	1,114	66,000	35,000	38,000	58%	28,000	42%	0	0%
	10	1,155	69,000	41,000	43,000	62%	26,000	38%	0	0%
	4	1,199	74,000	49,000	54,000	73%	19,000	26%	0	0%
**HC**	18	3,180	6,155,000	4,731,000	4,794,000	78%	0	0%	1,360,000	22%
	13	3,222	5,885,000	265,000	306,000	5%	531,000	9%	5,047,000	86%
	10	3,255	5,651,000	731,000	740,000	13%	225,000	4%	4,686,000	83%
	4	3,299	5,245,000	2,330,000	5,151,000	96%	0	0%	193,000	4%

Continental margin surface area is corrected for eustatic sea level rise [5, 6] and calculated using the SRTM30_PLUS global bathymetry dataset [50]. Total vertical extent of hypoxia is stated, including intermediate and severe. Hypoxic and oxic margin surface area (km^2^) and percent (%) are calculated for the seafloor above a region-specific isobaths: SP (0–3,300 mbsl), CC (0–2,400 mbsl), MM (1–1,200 mbsl), and HC (0–3,300 mbsl). Upper ocean oxic surface area (km^2^), from the surface ocean to the upper subsurface hypoxic boundary, is included.

Continental margin volume is corrected for eustatic sea level rise [5, 6] and calculated using the SRTM30_PLUS global bathymetry dataset [50]. Hypoxic and oxic margin volume (km^3^) and percent (%) are calculated for the water column above a region-specific isobath: SP (0–3,300 mbsl), CC (0–2,400 mbsl), BM (1–1,200 mbsl), and HC (1–3,300 mbsl). Upper ocean oxic volume (km^3^), from the surface ocean to the upper subsurface hypoxic boundary, is included.

## Results

### Subarctic Pacific

The SP is dominated at the surface by the eastward flowing North Pacific Current, which branches into the Alaskan Gyre and the southward-flowing CC (e.g., [52]). North Pacific Intermediate Water (NPIW) forms in the Sea of Okhotsk and mixes laterally into the Pacific subtropical gyre [53]. Deep water in the North Pacific is poorly ventilated, nutrient-rich and oxygen-depleted [18], and plays a critical role in maintaining extensive intermediate water OMZs [54, 55]. The SP has a modern, seasonal OMZ in the Gulf of Alaska (Fig. 2), attenuated in the summer, with the core of the OMZ from 670–1060 m in the water column [56]. Hypoxic waters spread westward, south of the Aleutian ridge and the Russian Kamchatka Peninsula, during winter months and form a seasonal OMZ that spans the Subarctic Pacific [56].

The SP is a region with inherent complexities, due to the influence of the Cordilleran Ice Sheet [57], the variability of sea ice formation [58], and circulation sensitivity to the episodic closures of the Bering Straight [59]. Previous investigations have characterized the Last Glacial Maximum (LGM) (Fig. 1) as a time of low surface ocean productivity, high ice-rafted debris sediment flux, and cold surface temperatures [60–65]. Archives track the deglacial oscillations of the warm Bølling-Allerød (B/A) and cool Younger Dryas (YD), consistent with the expected atmospheric teleconnections between the North Pacific and the North Atlantic [66]. The B/A and Holocene are warmer and more productive across the region, from the Gulf of Alaska [67] to the Western margin of Japan [68]. In step with the changes in surface productivity, seafloor hypoxia developed during the warm, productive intervals of the deglaciation across intermediate (600 mbsl, site RAMA 44PC) and deep (2,900 mbsl, site EW0408–85JC) water depths [58, 67–72]. The productivity and oxygenation oscillations of margin and shelf environments to Termination IA, the B/A and the YD reveal the substantial changes this contiguous high latitude environment recently underwent.

**Figure 5:**
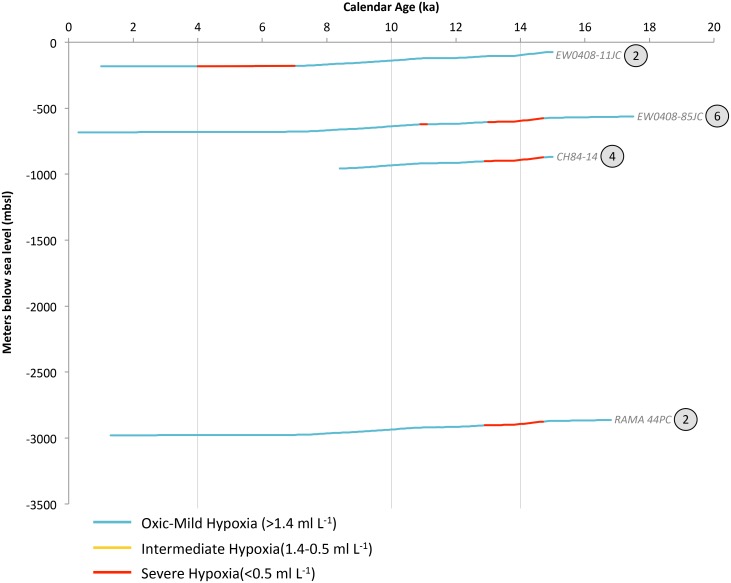
Subarctic Pacific (SP) deglacial core data synthesized into hypoxia categories. Changing deglacial core depths reflect global eustatic sea level change. The encircled number adjacent to each core label corresponds to the number of available oxygenation proxies, which are enumerate in Table 3. Vertical grey bars correlate to temporal intervals in OMZ geospatial reconstructions for this region.

**Figure 6:**
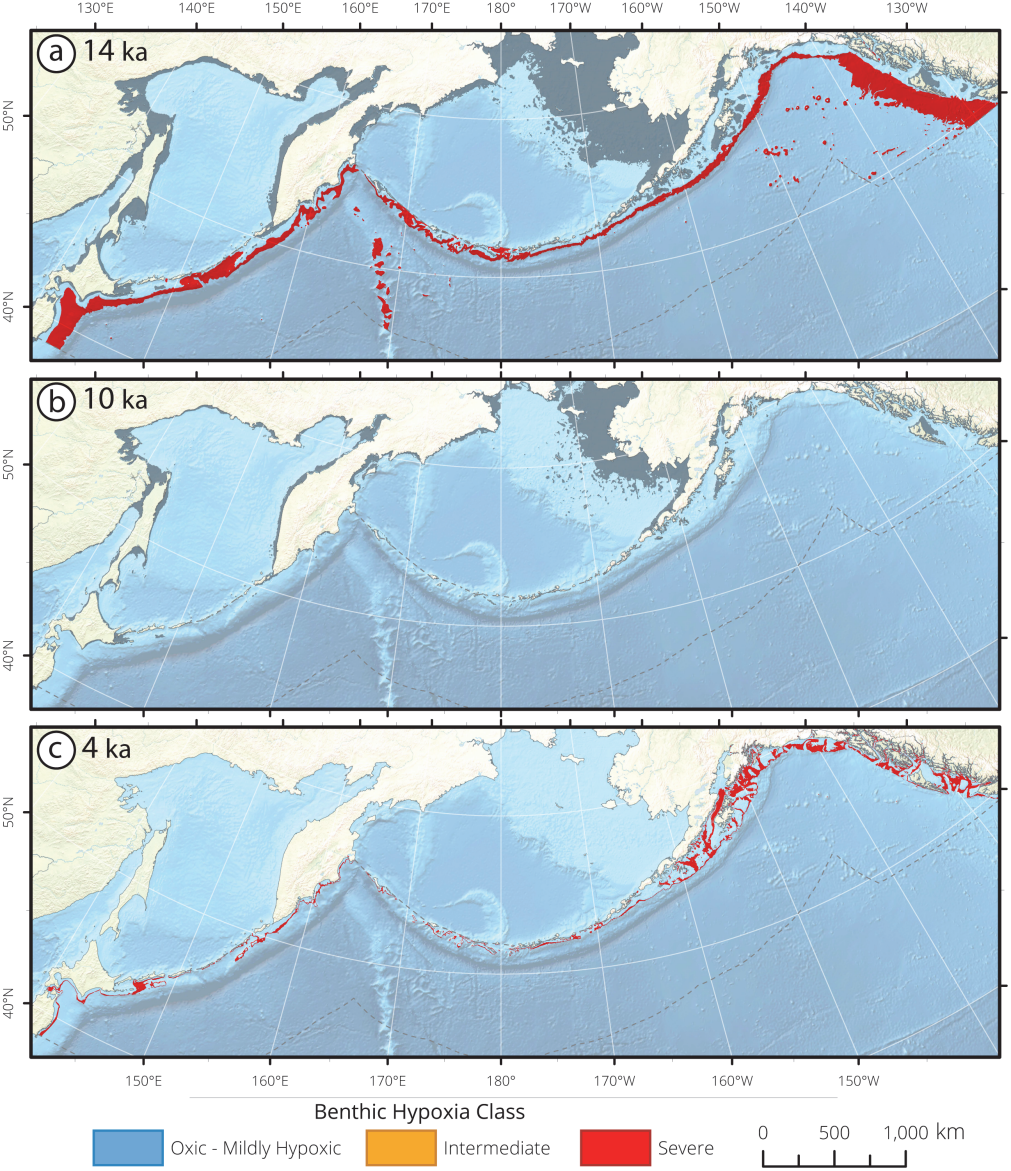
Subarctic Pacific bathymetric seafloor masks and surface area (km^2^) histograms of deglacial hypoxia impingement for (a) 14 ka, (b) 10 ka, and (c) 4 ka. The Bering Sea and Sea of Okhotsk are not included in the analysis, due to their unique, regional-scale oceanography. Seafloor is selected between 0–3,200 mbsl, with a southern latitude limit at 38° 30′ N in the Western Pacific and 49° 30′ N in the Eastern Pacific with a northern limit along the Aleutian Arc. Analyses were limited to the continental margin within a 400 nautical mile buffer offshore of the continental coastline and the Aleutian Arc. The changing gray shoreline through the panels depicts the paleo-shoreline. At 14 ka, severe hypoxia ranged from 596–2,894 mbsl. At 10 ka, the water column was oxygenated, and at 4 ka severe hypoxia was found between 132–232 mbsl.

**Paleoxygenation reconstructions for the Subarctic Pacific**. Four sediment cores meet the criteria for deglacial reconstructions: EW0408–85JC, EW0408–11JC, RAMA 44PC, and CH84–14 (Fig. 4; Table 3). Cores EW0408–85JC and EW0408–11JC were both collected from the Gulf of Alaska, RAMA 44PC was collected east of Kamchatka Peninsula, and CH84–14 was collected east of Hokkaidō Island, Japan. Shifts in seafloor oxygenation throughout the SP exhibit a cohesive, though limited, hydrographic picture (Figs. 5 and 6). We limit our SP reconstruction to mid-way through the deglaciation (14–4 ka), wherein the regional margin is extensively hypoxic, followed by a contraction to shallow, upper intermediate water hypoxia in the Holocene. The Bering Sea and Sea of Okhotsk are not included in the analysis, due to their unique, regional-scale oceanography.

Records from ODP Site 882 (Detroit Seamount, water depth 3244 mbsl) indicate that the deep glacial North Pacific was “suboxic” (based upon [U]) [73]. Although lacking a high enough sedimentation rate necessary to be included in this analysis, ODP Site 882 indicates that the deep SP (>3000 m) gained oxygen through the deglaciation. At Termination IA, the three deepest sites record the development of severe hypoxia, associated with laminations [67] and high authigenic [U] and [Mo] [74] in EW0408–85JC, and high sedimentary δ^15^N values in CH84–14 and RAMA 44PC [68]. These proxy records indicate that at 14 ka severe hypoxia ranged from 596–2,894 mbsl (Fig. 5). Regional hypoxia was followed by subsequent oxygenation in intermediate and deep waters through the YD, Termination 1B and the early Holocene. In the mid-Holocene, the shallowest site recorded the development of severe hypoxia in upper intermediate waters ([Mo]) [74].

Paleoceanographic reconstructions from the SP indicate extensive and severe hypoxia developed during the warming of the deglaciation, and suggest severe hypoxia as shallow as ~600 mbsl and across ~2,298 m of contiguously hypoxic water column (Fig. 6). The absence of hypoxia during the YD cooling of the Northern Hemisphere indicates that the subsurface SP is sensitive to rapid climatic oscillations. Coherent oxygenation oscillations, from the Gulf of Alaska to Eastern Japan, indicate that geographically widespread changes occur in response to global climate change.

### California Current

The CC is the eastern limb of the North Pacific Subtropical Gyre (e.g., [75–77]), and is driven onshore in the Northern Hemisphere spring [54]. The CC is characterized by seasonal upwelling cycles, a highly productive continental margin, and an acute and extensive subsurface OMZ [54, 55, 78]. Additionally, a strong nearshore, poleward Counter Current, know as the Davidson Counter Current, has a surface and subsurface expression [79] and brings warmer, saltier, less oxygenated, and equatorially-influenced water up the continental margin. The CC OMZ exhibits an onshore-offshore gradient, wherein the lowest oxygen concentration values are found within 200 km from the coastline [54]. The upper boundary of the CC OMZ (as defined by [O_2_ = 1.4 ml L^-1^) is relatively deep (~600 mbsl), and the OMZ is thick (~1200–1500 m) (Fig. 1). Oxygen delivery to the CC subsurface is balanced by contributions from well-oxygenated, northern-sourced NPIW and poorly oxygenated, southern-sourced intermediate waters [53].

Early investigations into CC continental margin sediments described the presence of laminations during the late Pleistocene to Holocene transition [80–82]. ODP Site 893A (576.5 mbsl) stands out amongst the region’s extensive suite of cores [27, 83–88] as a globally recognized site with high-resolution synchroneity to Northern and Southern Hemisphere climate events [40, 88, 89]. The expansion and contraction of the regional CC OMZ occurred with remarkable fidelity to the glacial terminations, warming intervals, and cooling oscillations of the Northern Hemisphere, with clear regional-scale deoxygenation associated with Termination IA, the B/A, Termination IB and the start of the Holocene (e.g., [27, 29, 40, 42, 90, 91]). Sediment cores from Vancouver Island record OMZ expansion from 13.5 to 12.6 ka, slightly delayed from more southern CC sites [92].

**Paleoxygenation Reconstructions for the California Current**. Eleven deglacial sediment cores were included in our geospatial analysis, from Santa Barbara Basin (ODP Site 893A, MD02–2504, MD02–2504, MV0811–15JC), Pt. Conception (ODP Site 1017E), Central California (F-8–90-G21, F2–92-P3, F2–92-P40, F2–92-P34), the Oregon-California border (ODP Site 1019E), and Vancouver Island (JT96–09) (Fig. 4, Table 3). These data indicate the extensive and shallow influence of OMZ waters on the CC continental margin during the deglaciation, and the regional-scale sensitivity of abrupt hydrographic change to Northern Hemisphere rapid warming and cooling events.

**Figure 7:**
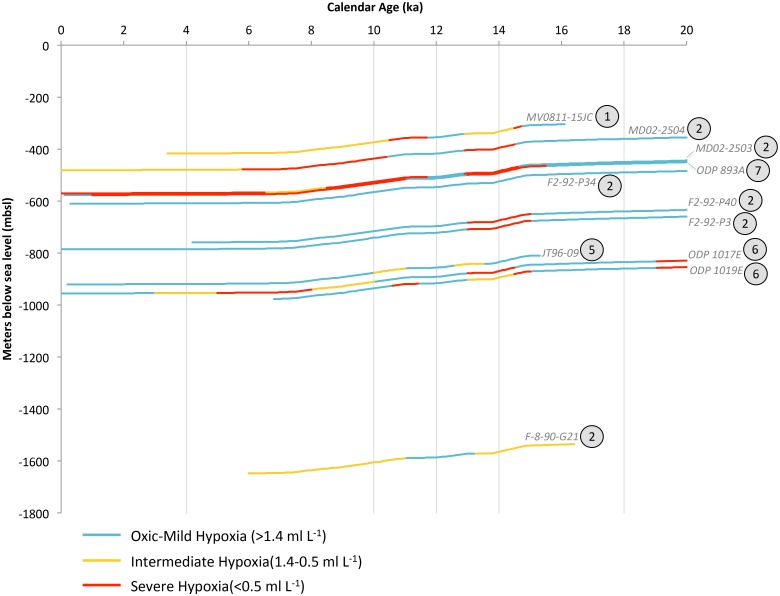
California Current (CC) deglacial core data synthesized into hypoxia categories. Changing deglacial core depths reflect global eustatic sea level change. The encircled number adjacent to each core label corresponds to the number of available oxygenation proxies, which are enumerate in Table 3. Vertical grey bars correlate to temporal intervals in OMZ geospatial reconstructions for this region.

Intermediate waters (>1,000 mbsl) in the CC were oxygenated during the LGM, up until Termination IA at 14.7 ka [27, 29, 40, 88, 93, 94]. The three deepest sites exhibit evidence of hypoxia in the LGM, including ODP Site 1017E and ODP Site 1019E, and F-8–90-G21 (Fig. 7). The ODP cores have elevated [Mo] values in the LGM [42], and hypoxia-associated foraminiferan marker species are preserved in the deepest core [95–97].

**Figure 8:**
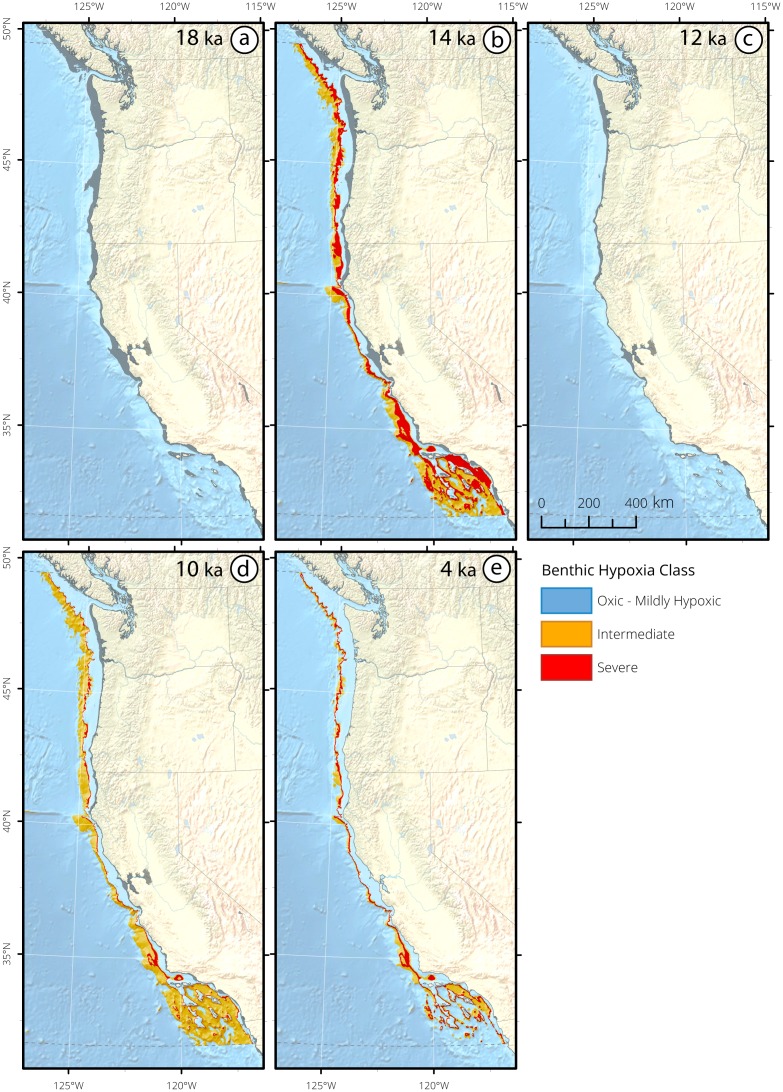
California Current bathymetric seafloor masks and surface area (km^2^) histograms of deglacial hypoxia impingement for (a) 18 ka, (b) 14 ka, (c) 12 ka, (d) 10 ka, and (e) 4 ka. Seafloor is selected between 0–2,400 mbsl and latitudinally constrained between 31° 40′-49° 30′ N. Analyses were limited to the continental margin within a 400 nautical mile buffer offshore of the continental coastline. The changing gray shoreline through the panels depicts the paleo-shoreline. The seafloor was oxic at 18 ka. At 14 ka the seafloor was severely hypoxic (395–869 mbsl), and bracketed by intermediate hypoxia above (332–395 mbsl) and below (869–1,564 mbsl). The seafloor returned to an oxic state at 12 ka. At 10 ka severe hypoxia (436–525 mbsl) was bracketed by intermediate hypoxia (373–436 mbsl, 525–1,605 mbsl). At 4 ka severe hypoxia (525–625 mbsl) was bracketed by intermediate hypoxia (417–525 mbsl,625–954 mbsl).

Geochemical ([Mo], %C_org_, and δ^15^N) data for all ODP Sites in the CC region, including Santa Barbara [42, 98–100], Pt. Conception [94], and the California/Oregon border [42, 101] collectively reconstruct the presence of denitrifying and severely hypoxic waters along the California margin at Termination 1A and through the Bølling-Allerød (Fig. 7). The Santa Barbara Basin sites record the OMZ intensification as laminations, severely hypoxic foraminiferan communities, and elevated redox metal concentrations [29, 40, 89, 93, 102, 103]. Archives from central California margin exhibit preserved laminations and elevated concentrations of [Mo] and [Cd] [40, 95, 96, 104, 105]. Core F2–92-P34 is anomalous to the broad CC pattern, with no enrichment of [Mo] at Termination IA and only a slight increase in %C_org_ [105]. At 14 ka, the records in this region indicate the seafloor was severely hypoxic from 395–869 mbsl and was bracketed above (332–395 mbsl) and below (869–1,564 mbsl) by intermediate hypoxia (Fig. 8).

Intermediate waters returned to oxygenated conditions midway through the deglaciation (12.9–11.7 ka), synchronous with the ephemeral YD cooling observed in other Northern Hemisphere climate records (Fig. 8) [106, 107]. This margin-wide oxygenation is evidenced by the unanimous absence of laminations across all depths, foraminiferal assemblages that reflect increased oxygen concentrations, and low concentrations of redox metals in all regional cores. Termination IB (11.7 ka) initiated a return to regional hypoxia, detected in eight of the cores. Sites within the Santa Barbara Basin preserved laminations at Termination IB [29] and were dominated by hypoxic, OMZ-associated foraminiferal communities [93, 102, 103]. Termination IB was a secondary expansion of the CC OMZ, and is associated with a narrow band of severely hypoxic water at 436–525 mbsl, bracketed by intermediate hypoxia from 417–525 mbsl and 625–954 mbsl (Fig. 8).

Paleoceanographic reconstructions from the CC reveal the extraordinary shallowness (severe hypoxia <300 mbsl) and extensity (1,233 m of contiguously hypoxic water column) of the regional OMZ during the recent deglaciation. Oxygenated upper ocean ecosystems are dramatically compressed at both Termination events to <300 m from the ocean surface. The YD cooling mid-way through the deglaciation is a remarkably ephemeral event of regional oxygenation. The analysis presented here reveals how geographically pervasive and temporally responsive subsurface oxygen concentrations in the CC system are to global climate change.

### Mexico Margin

The MM OMZ is remarkable in thickness and intensity (Fig. 2), and is a product of high surface production, a sharp pycnocline that inhibits local ventilation of subsurface waters, and the “sluggish and convoluted deep circulation” of regional subpycnocline waters [108]. The eastern Pacific sea surface temperature warm pool is centered off of southern Mexico and Guatemala, a product of the large seasonal net heat flux and weak wind mixing of the region [108, 109]. The CC and NPIW travel equatorward along Baja California and at 25° N turn westward with gyral circulation [110–112], maintaining an oceanographic transition zone between 20°-30° N. In the north, the OMZ sits between 500–1,000 mbsl [56] and in the south shoals upwards (100–700 mbsl), due to the influence of southern sourced intermediate water (including AAIW and “Equatorial Waters”) [113]. The oxygen concentration of these waters is low (~0.2 ml L^-1^), and the upper hypoxic boundary is extremely shallow. The term “Equatorial Waters” refers to a composite of Subtropical Underwater found below the equator [114, 115], north and south subtropical surface water [108], 13°C waters [116, 117] and subtropical mode water [118].

Sediment records in this region indicate major reductions in bottom water oxygenation through the last deglaciation, however the deglacial intensification of the OMZ does not appear to be synchronous across multiple locations [16]. Primary production and carbon export is thought to have been low during the LGM [119], potentially due to reduced upwelling [17, 120]. Changes in the depth of the equatorial thermocline and nutricline have been hypothesized to drive deglacial oscillations in oxygenation [121]. Unlike SP and CC records, oxygenation oscillations are not a comprehensive feature of the margin record, and only appear in records from the Sea of Cortez, which is isolated from the open margin and considered a more continental record of surface and atmospheric conditions [16, 26]. The equatorial Pacific is a major global denitrification zone. δ^15^N records from MM sites exhibit glacial-interglacial variability [122] and synchroneity to sites along the Peru-Chile margin [123–125].

**Figure 9:**
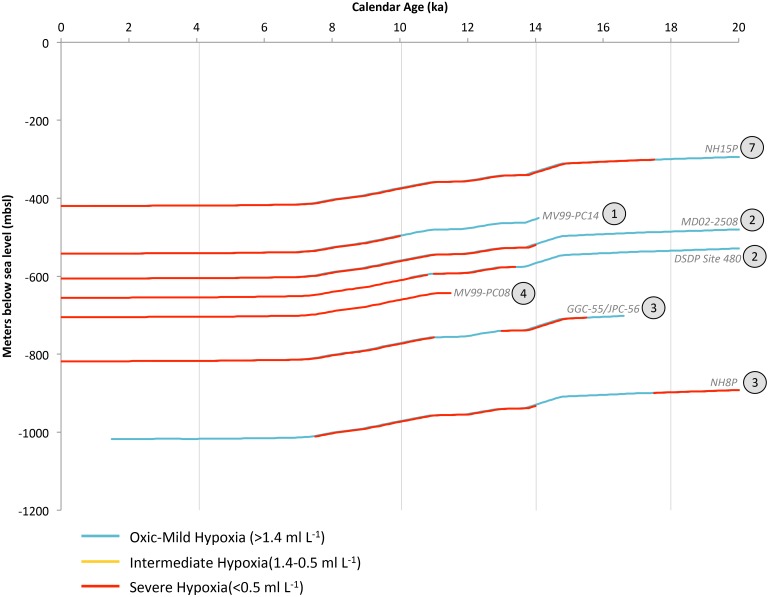
Mexico Margin (MM) deglacial core data synthesized into hypoxia categories. Changing deglacial core depths reflect global eustatic sea level change. The encircled number adjacent to each core label corresponds to the number of available oxygenation proxies, which are enumerate in Table 3. Vertical grey bars correlate to temporal intervals in OMZ geospatial reconstructions for this region.

**Paleoxygenation Reconstructions for the Mexico Margin**. Seven paleoceanographic records were selected for deglacial reconstructions, including two cores within the Sea of Cortez (GGC-55/JPC-56 and DSDP Site 480), three cores along the southwestern margin of Baja California Sur (MV99-PC14, MV99-PC08, MD02–2508 and GC31/PC08) and two cores along the Mexican Margin (NH8P, NH15P) (Fig. 4; Table 4). The MM exhibits reduced regional coherency, with asynchronous hypoxia and oxygenation oscillations (Fig. 9). However, this region exhibits a clear trend of higher oxygenation in the LGM, transitioning to severe hypoxia in intermediate waters at the start of the Holocene.

The two deepest, southernmost sites from the Mexican margin suggest the seafloor was hypoxic during the LGM, as indicated by the presence of laminations (Site NH8P) [126, 127] and high [Mo] concentrations (Site NH22P) [119]. At 18 ka, hypoxia was detected at only one site in intermediate waters (898 mbsl). Hypoxic waters spatially expanded along the Mexican margin and in the Sea of Cortez at 14 ka, and ranged from 334–932 mbsl. Site NH8P records the expansion of severe hypoxia along the margin, with laminations preserved between 15–7.5 ka [126, 127]. Site GGC-55/JPC-56 preserved laminations from 14.8–12.5 ka, concomitant with high δ^15^N values (>14‰), and high opal accumulations rates [128]. Lamination preservation occurred from 13–11.2 ka in DSDP Site 480, subsequently followed by bioturbation from 11.2–10.5 ka [26].

**Figure 10:**
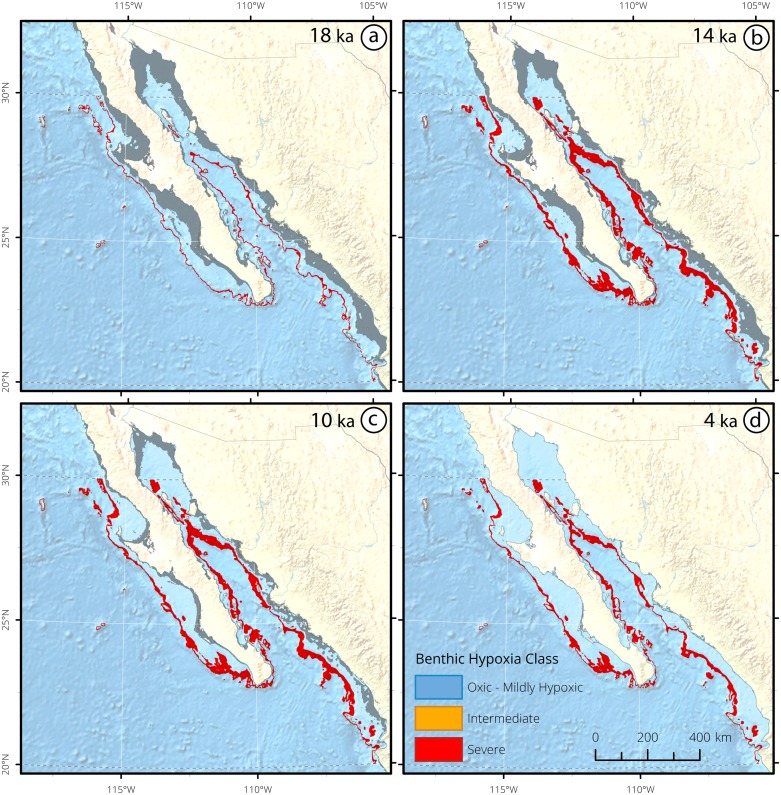
Mexico Margin bathymetric seafloor masks and surface area (km^2^) histograms of deglacial hypoxia impingement for (a) 18 ka, (b) 14 ka, (c) 10 ka, and (d) 4 ka. Seafloor is selected between 0–1,200 mbsl and latitudinally constrained between 20°-30° N. Analyses were limited to the continental margin within a 400 nautical mile buffer offshore of the continental coastline. The changing gray shoreline through the panels depicts the paleo-shoreline. At 18 ka, severe hypoxia was limited from 937–1,037 mbsl. Severe hypoxia ranged from 334–932 mbsl at 14 ka. At 10 ka, severe hypoxia was found from 375–973 mbsl, and at 4 ka severe hypoxia was contracted slightly to 419–817 mbsl.

Five sites, including MV99-PC14, MD02–2508, DSDP 480, GC31/PC08, and GGC-55/JPC-56 (all from Sea of Cortez and Western Baja), recorded a synchronous northward expansion of hypoxic intermediate waters at ~11–10 ka (Fig. 10), with severe hypoxia extending from 375–973 mbsl. The regional shift was recorded in laminations at DSDP Site 480 [128]. At the southern tip of Baja (GC31/PC08 and MD02–2508), lamination preservation and steep increases in redox metals occurred [16, 17, 105, 121, 129]. Core MV99-PC14, extracted from Soledad Basin (which is oxygenated by sill depth waters at 290 mbsl) [16], became strongly laminated at 10 ka, indicating that extremely shallow upper intermediate waters deoxygenated at the start of the Holocene. The Holocene remained severely hypoxic at 4 ka from 419–817 mbsl.

Paleoceanographic reconstructions for MM provide evidence for the regional intensification of subsurface hypoxia through the recent deglaciation. The MM exhibited reduced sensitivity (Fig. 10) to the first Northern Hemisphere glacial termination event (14.7 ka), as compared to the NP (Fig. 6) and CC sites (Fig. 8). A regional-scale expansion of hypoxia was recorded at ~10–11 ka, wherein ~600 m of the water column became severely hypoxic (Figs. 9 and 10).

### Eastern Equatorial Pacific and Humboldt Current

The HC, also known as the Peru Current, is the eastern limb of the South Pacific subtropical gyre, characterized by upwelling and extreme biological productivity [130]. A thick (~500 m), intense ([O_2_]<0.2 ml L^-1^) and shallow (upper boundary ~50 mbsl) OMZ characterizes the HC (Fig. 2)[131]. Cold HC surface waters move north along the South American continental margin, and then become a part of the equatorial cold tongue. The southward-flowing Peru-Chile Undercurrent is found 75–500 mbsl, in association with the OMZ [132]. This equatorially-derived water is transported as far as 48° S [133], and is derived from Equatorial 13°C Waters [116, 117], Subtropical Underwater [115], and Eastern South Pacific Intermediate Water [134]. The spatial distribution of OMZ thickness is correlated with upwelling conditions [135]. An intense upwelling system is located off of Peru [136, 137], which brings nutrient-rich AAIW to the surface and stimulates high production in the equatorial cold tongue [138–140]. A more seasonal upwelling cell exists southward off of Chile [141]. A functional break in deep water properties exists at 15–25° S, where tropical and subtropical deep waters are dynamically separated, as deep waters are directly connected in the Western Pacific [142]. Abyssal waters (>4,000 m) are a mixture of water from the Weddell Sea and the North Atlantic, and are relatively high in oxygen concentrations [143].

The deglaciation is thought to have co-occurred with extensive ventilation of the deep-sea, renewing oxygen concentrations in the deep ocean interior (e.g., [2, 63, 73, 144]). Recent syntheses document global increases in deep ocean [O_2_] through the deglaciation, reflecting the transfer of respired carbon from the deep ocean to the atmospheric and surface ocean carbon pool [7]. Numerous high quality records of deep-sea oxygenation are present in the Eastern Equatorial Pacific, and are included in the HC analysis. Intermediate water records from the HC are limited; however, it is clear that intermediate water (~300–1,400 mbsl) deoxygenated at these depths prior to the Northern Hemisphere glacial Terminations [124, 145].

**Figure 11:**
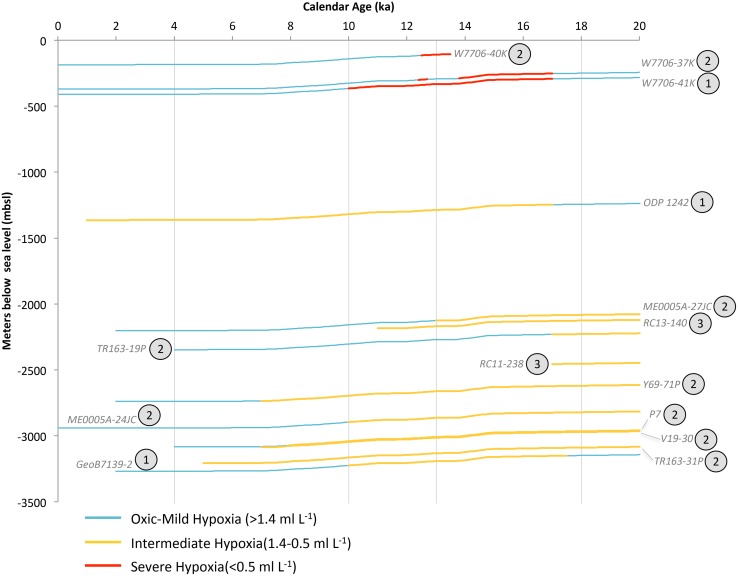
Equatorial Pacific and Humboldt Current (HC) deglacial core data synthesized into hypoxia categories. Changing deglacial core depths reflect global eustatic sea level change. The encircled number adjacent to each core label corresponds to the number of available oxygenation proxies, which are enumerate in Table 3. Vertical grey bars correlate to temporal intervals in OMZ geospatial reconstructions for this region.

**Figure 12:**
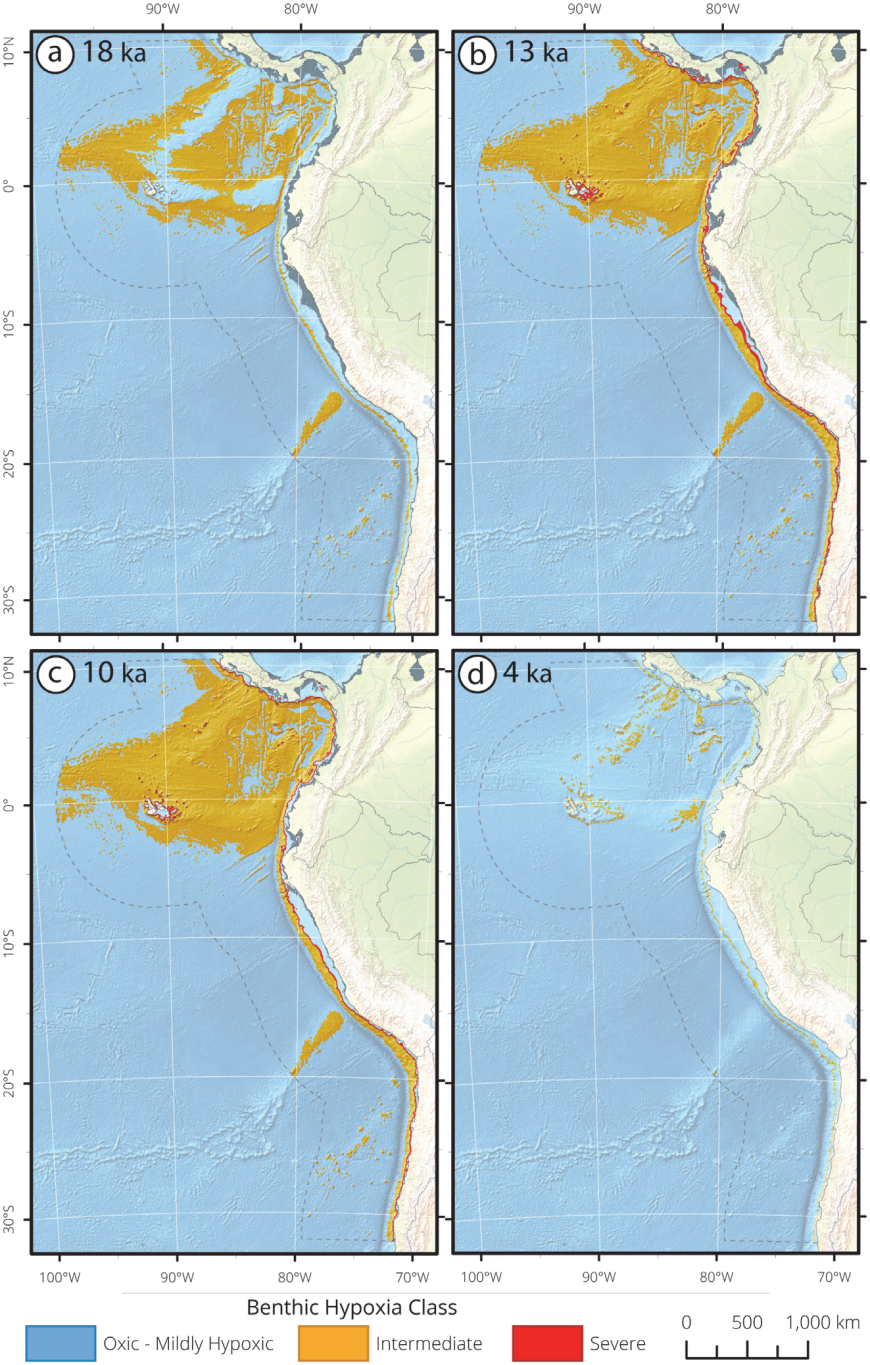
Equatorial Pacific and Humboldt Current bathymetric seafloor masks and surface area (km^2^) histograms of deglacial hypoxia impingement for (a) 18 ka, (b) 13 ka, (c) 10 ka, and (d) 4 ka. Seafloor is selected between 0–3,300 mbsl and latitudinally constrained between 10° 30′ N-32° S. Analyses were limited to the continental margin within a 400 nautical mile buffer offshore of the continental coastline and the Galapagos Islands. The changing gray shoreline through the panels depicts the paleo-shoreline. At 18 ka, severe hypoxia was limited from 937–1,037 mbsl. At 18 ka, intermediate hypoxia was found in deep water (2,082–3,088 mbsl). At 13 ka, severe hypoxia was found between 108–331 mbsl, and intermediate hypoxia was deeper (332–3,130 mbsl). At 10 ka, severe hypoxia was again found in shallow waters (315–415 mbsl) and intermediate hypoxia was deeper (415–3,164 mbsl). At 4 ka, intermediate hypoxia retracted to between 1,313–1,413 mbsl.

**Paleoxygenation reconstructions for the Equatorial Pacific and Humboldt Current**. Thirteen paleoceanographic cores met the criteria for deglacial geospatial reconstructions, including cores from the Panamanian margin (ODP Site 1242), the Galapagos (TR1–163–19P, RC13–140, Y69–71P, ME0005A-24JC, RC11–238), Ecuador (P7, ME0005A-27JC), Peru (V19–30, TR163–31P, W7706–41K, W7706–40K, W7706–37K), and the Chilean margin (GeoB7139–2) (Fig. 4; Table 4). Sites exhibit regional synchroneity from deep to upper intermediate water depths, lack coherent timing with Northern Hemisphere climate records (Fig. 11), and exhibit a deglacial temporal signature similar to the Southern Hemisphere (e.g., [146, 147]). Broad deglacial patterns include increasing deep ocean oxygenation, severe and shallow hypoxia shoaling at ~17 ka in upper intermediate water, and a ~6 kya temporal overlap between deep and intermediate hypoxia (Fig. 12).

For the eight equatorial deep waters sites, from 2,203–3,091 mbsl, the LGM is associated with high concentrations of [U] [144, 148, 149]. These records reconstruct the presence of intermediate hypoxia at 18 ka from 2,082–3,088 mbsl. The deepest, most southerly record (GeoB7139–2) stands out as anomalous to the deep equatorial cores, wherein δ^15^N values rapidly increased (~5–6‰) at ~17 ka, suggesting a shift towards denitrification and an associated decrease in oxygen concentration [123, 150]. Deep-sea hypoxia continues to be a regional feature through the deglaciation and into the mid-Holocene, although the timing of post-glacial deep ocean oxygenation is debated. Concentrations of [U] remain high into the mid-Holocene in the equatorial cores [144], beyond 14.7 ka, which differs from the timing of oxygenation in the North Pacific [73]. This temporal difference may be an artifact of increases in productivity and organic matter flux, or it may be a true signal of the temporal differences in oxygenation across the Pacific. For our purposes here, we follow existing interpretations of high [U] in the post-glacial Equatorial Pacific as a signal of intermediate hypoxia (Fig. 11).

Deoxygenation in intermediate waters occurred at ~17 ka, as exhibited by laminations, a ~5‰ δ^15^N increase along the Chilean Margin (W7706–41K, W7706–40K, W7706–37K) [145, 151], and high δ^15^N values adjacent to the Panamanian Margin (ODP Site 1242; Fig. 11) [124]. Sedimentation is discontinuous through the deglaciation in W7706–37K and may be due to sediment disturbance rather than a signal of oxygenation reversal [145]. Together, these cores indicate severe hypoxia at 13 ka ranged from 108–331 mbsl and intermediate hypoxia ranged from 332–3,130 mbsl. At 10 ka, severe hypoxia is recorded in upper intermediate waters (365±50 mbsl), and is bordered below by intermediate hypoxia from 415–3,164 mbsl. At 4 ka, sediment at the deep equatorial sites shows dramatic reductions in [U] concentrations. Concurrently, the shallow central Chilean sites were oxygenated, and the remaining location with a clear hypoxia signal is limited to Panamanian ODP Site 1242. As such, hypoxia at 4 ka is substantially attenuated to 1,363 mbsl.

The HC provides a unique record of synoptic changes occurring in both deep and intermediate water through the events of the deglaciation. The HC exhibits a coherent regional signal of oxygenation, while lacking synchroneity to the Northern Hemisphere. Striking features of the record include the regional deoxygenation of upper intermediate waters at 17 ka, the extreme vertical expansion of hypoxic water at 13 ka, and the ~6 kyr overlap of deep and intermediate hypoxia creating ~3,000 m of contiguous hypoxic water column.

### Benguela Current

The BC system, also referred to as the Angola-Benguela Current, is the equatorward flowing Eastern Boundary Current of the South Atlantic subtropical Gyre (Fig. 2), associated with high productivity, organic-rich sediments, and seasonal upwelling [152]. Upwelling events, and the export of surface productivity, are linked directly to modern subsurface OMZ structure [153, 154]. The HC OMZ is significantly shallower and less geographically extensive than other systems reviewed here (Fig. 2). The core of the BC OMZ is between 300–400 mbsl, and hypoxic waters extend to as shallow as 50 m [155].

Sediment records from the BC reveal mixed deglacial productivity signals, wherein a few single sites indicate decreased productivity during the LGM [156], while more recent and contradictory work indicates a decrease in surface productivity in the Holocene as compared to the LGM [157–160]. Contributing to this mixed signal, it appears that the productivity center may have moved offshore since the LGM [160]. From the evidence available, it does not appear that the OMZ associated with the BC follows a glacial/interglacial cycle like that which dominates the Pacific, but is both more heterogeneous and directly linked to regional cycles of productivity and upwelling [160].

### Oman and Pakistan Margin

The OPM, within the Indian Ocean, has the most globally extensive OMZ in terms of water column vertical extent (>1000 m) [161] (Fig. 2). Subsurface dissolved oxygen content of the region is controlled by nutrient additions, changes in intermediate and deep water ventilation, and convective mixing of deep waters [162]. Oxygen depleted waters sourced from small inlet seas contribute to the maintenance of severe hypoxia in the region [163]. Monsoonal seasonality of the Arabian Sea drives OMZ oscillations, wherein strong onshore winds force the upwelling of nutrient-rich waters [164, 165]. OPM sediments are more organic rich than other low-latitude OMZ regions, attributed to high primary productivity and reduced remineralization at depth [166].

The OPM is a sensitive recorder of climate variability and an archive of the connectivity between low latitude monsoonal dynamics and high latitude temperature variability [167, 168]. Paleoceanographic investigations show that the strength of the OPM monsoon has high-resolution, sub-millennial cycles that exhibit synchrony to Greenland climate variability [168]. Regional surface water productivity has co-varied with OMZ intensity, such that OMZ intensity is especially weak during cold stadial events when summer monsoon effects are reduced [167, 169–172], while OMZ intensity is enhanced during warm interstadials (e.g., the B/A) [171]. Deglacial cores demonstrate rapid (10^2^ year timescales) shifts from intermediate to severe hypoxia due to climate forcing [173]. Weakening of the OMZ occurred during Heinrich 1 (~18–14 ka) and the YD [174, 175]. Monsoon intensity peaked with a corresponding increase in surface productivity [172, 176] and ocean hypoxia from 9.5 to 5.5 ka [177, 178]. Geospatial reconstructions of deglacial hypoxia were not conducted for the OPM, as the limited high-resolution deglacial records were not sufficient to warrant further analyses.

## Discussion

### Mechanisms and implications of OMZ variation

Large-scale physical and biogeochemical processes in the ocean drive OMZ formation, and have high-latitude and low-latitude derived features. As such, the deglacial changes in dissolved oxygen may be explained by proximal and distal mechanisms. High latitude sources for deglacial OMZ variability have been hypothesized to include changes in intermediate water production/ventilation [17, 27, 40, 68, 71, 84, 93, 150, 179] and changes in deep ocean circulation [70, 123, 170]. Deglacial micronutrient [Fe] enrichment has been hypothesized to intensify hypoxia, particularly in the Subarctic Pacific [58, 67, 151]. Additionally, oxygen consumption at the site of NPIW subduction has been hypothesized as a high latitude mechanism [68]. Low latitude mechanisms for deglacial OMZ variability include the relative importance of equatorial counter currents [87, 122], the intensity and location of upwelling systems [127, 180] changes in the oceanic preformed nutrient inventory [73, 124, 126, 128, 181], surface ocean productivity [119, 121], atmospheric structural changes [17, 85, 182], and the strength of monsoonal systems [167, 168].

In some cases, independence between mechanisms that force hypoxia has been demonstrated, for example where local productivity and the high-latitude ventilation of intermediate waters are shown to decouple during the deglaciation [90, 183]. However, many of the physical and biological processes that drive the development of subsurface hypoxia are ultimately linked. Indeed, circulation models from the latter half of the 20^th^ century have revealed that changes in the rates of surface productivity can ultimately be driven by physical perturbations in circulation, through interactions between thermocline depth, nutrient flux, and particulate export into hypoxic waters [184].

Major changes in the distribution of [O_2_] in Eastern Boundary Currents occurred during the recent deglaciation. The substantial oxygen changes arose concomitantly with increasing atmospheric carbon concentration, surface warming and rising sea levels [2–6]. The analyses presented here reveal that rapid oscillations in oxygen distribution are an inherent feature of large ocean regions, as subsurface [O_2_] exhibits extreme sensitivity to hemisphere-specific warming and cooling. Mid-way through the deglaciation, hypoxic waters shoaled vertically, shifting the upper OMZ boundary towards the ocean surface, with shoaled depths ranging from 596 mbsl in the SP (Fig. 6), 332 mbsl in the CC (Fig. 8), 334 in the MM (Fig. 10), to 108 mbsl in the HC (Fig. 12). Subsurface hypoxia expanded and intensified, creating OMZ environments which ranged in thickness from 2,298 m in the SP, 1,233 m in the CC, 598 m in the MM, to 3,022 m in the HC (Table 4). These data provide evidence of the capacity for OMZs to exhibit extreme shallowness and water column extensity to states that have no analogue in the modern ocean.

The deglaciation offers an extremely informative case study of the sensitivity and coupling between OMZs and global climate oscillations. Our analyses show that during global-scale warming events, vast expanses of the upper global ocean deoxygenate, resulting in the vertical compression of oxygenated ocean ecosystems. These analyses of past changes in dissolved oxygen are fundamentally relevant to the loss of [O_2_] observed in the modern ocean [185–187], and provide bounds and reasonable expectations for expanding OMZs in the future.

Changes in the distribution of oxygen translate directly to the structure of marine ecosystems (e.g., [11, 12]) and OMZ expansions have implications for modern oceanography, biodiversity conservation, ocean management and sustainable fisheries. Modern oceanographers can anticipate that Eastern Boundary Current OMZs have the capacity to expand to hydrographic structures that have never been instrumentally observed. These analyses underscore the continued need for high-quality instrumental hypoxic time series data and predictive models of modern oceanographic change. The challenge to resource managers and conservationists is how to plan for the great deal of uncertainty introduced when ecosystems undergo changes that only have precedent in the geologic record.

### Modern subsurface oxygen variability

In the modern ocean, the vertical expansion and intensification of OMZ regions has been detected in the California Current [185, 188], equatorial waters [186], the Subarctic [189, 190] and Subtropical Pacific [191], the North Atlantic [192], the Indian Ocean [193] and the Southern Ocean [194]. The loss of subsurface dissolved oxygen is an acute perturbation to coastal ecosystems and both benthic and pelagic communities [187]. These data indicate that deoxygenation in the 20^th^ and 21^st^ Centuries is a feature of every global ocean basin. However, unique physical and biological processes within oceanographic provinces provide a more nuanced and complicated view of climate-forced ocean deoxygenation.

OMZs exhibit high-frequency variability on diurnal and semi-diurnal timescales [195], on the intra-annual timescales of upwelling and relaxation cycles [196], and in tight association in the California Current with La Niña events [197]. These OMZ oscillations illustrate the potential for undescribed scales of variability in the coastal ocean. Additionally, as longer instrumental time series are developed, novel questions regarding intrinsic high-frequency oceanographic variability arise. For example, conflicting interpretations of California Current oxygen trends exist, such that the data can be analyzed to reveal a long-term deoxygenation trend [185] or a 20–25 year undescribed oscillation [188]. This interpretive conflict highlights the importance of understanding high frequency variability of oxygen concentrations, which may be overprinted by anthropogenic climate forcing. Complicating the picture even more so, recent analyses of historical sediments from the Eastern Equatorial Pacific indicate that the regional OMZ has contracted, and that this contraction is correlated with the slackening of trade winds in the tropical Pacific [198]. These regional trends in dissolved oxygen illustrate the potential for OMZ oceanographic provinces to respond differently to anthropogenic climate forcing, however 20^th^ century oxygenation in the Equatorial Pacific is thought to be anomalous in the context of a broader global picture. On centennial time scales, processes associated with a warm surface ocean, including gas solubility reduction and physical stratification, are predicted to substantially reduce dissolved oxygen concentrations in the ocean interior [8, 184].

## Conclusions

We integrate existing deglacial geochemical, sedimentary, and microfossil oxygenation proxies to reconstruct the timing, depth and intensity of seafloor hypoxia in Eastern Boundary Currents, principally in the Eastern Pacific. These analyses illustrate the high degree of coupling between the global climate system and OMZ environments and provide the most comprehensive window, to date, into the spatial capacity of OMZ ecosystems to expand and contract due to climate change. The recent deglaciation was accompanied by the dramatic shoaling of the upper hypoxic boundary toward the ocean surface, the compression of upper ocean oxygenated habitat, and the expansion of the subsurface hypoxic water column. Subarctic Pacific and California Current continental margins exhibit tight correlation to the oscillations of Northern Hemisphere deglacial events, whereas the Mexico Margin and the Equatorial Pacific and Humboldt Current exhibit hypoxia expansion prior to Termination IA (14.7 ka), and no regional oxygenation oscillations. Oxygenation changes occurred in synchrony across ocean basins, revealing the extensive sensitivity of upper ocean systems to changes in global climate.

## References

[1] Alley RB (1999) The deglaciation of the Northern Hemisphere: A Global Perspective. Annual Review of Earth and Planetary Sciences Letters 27: 149–182. 10.1146/annurev.earth.27.1.149

[2] Sigman DM, Boyle EA (2000) Glacial/interglacial variations in atmospheric carbon dioxide. Nature 407: 859–869. 10.1038/35038000 11057657

[3] Monnin E, Indermühle A, Dällenbach A, Flückiger J, Stauffer B, et al (2001) Atmospheric CO_2_ concentrations over the last glacial termination. Science 291: 112–114. 10.1126/science.291.5501.112 11141559

[4] Shakun JD, Clark PU, He F, Marcott SA, Mix AC, et al (2012) Global warming preceded by increasing carbon dioxide concentrations during the last deglaciation. Nature 484: 49–54. 10.1038/nature10915 22481357

[5] Fleming K, Johnston P, Zwartz D, Yokoyama Y, Lambeck K, et al (1998) Refining the eustatic sea-level curve since the Last Glacial Maximum using far- and intermediate-field sites. Earth and Planetary Science Letters 163: 327–342. 10.1016/S0012-821X(98)00198-8

[6] Milne GA, Long AJ, Bassett SE (2005) Modeling Holocene relative sea-level observations from the Caribbean and South America. Quaternary Science Reviews 24: 1183–1201. 10.1016/j.quascirev.2004.10.005

[7] Jaccard SL, Galbraith ED (2012) Large climate-driven changes of oceanic oxygen concentrations during the last deglaciation. Nature Geoscience 5: 151–156. 10.1038/ngeo1352

[8] Keeling RF, Oschlies A, Orr JC (2009) Atmospheric evidence for recent global ocean deoxygenation. Geochimica et Cosmochimica Acta 73: A632–A632.

[9] Oschlies A, Schulz KG, Riebesell U, Schmittner A (2008) Simulated 21^st^ century’s increase in oceanic suboxia by CO_2_-enhanced biotic carbon export. Global Biogeochemical Cycles 22 10.1029/2007GB003147

[10] Shaffer G, Olsen SM, Pedersen JOP (2009) Long-term ocean oxygen depletion in response to carbon dioxide emissions from fossil fuels. Nature Geoscience 2: 105–109. 10.1038/ngeo420

[11] Zhang J, Gilbert D, Gooday AJ, Levin L, Naqvi SWA, et al (2010) Natural and human-induced hypoxia and consequences for coastal areas: synthesis and future development. Biogeosciences 7: 1443–1467. 10.5194/bg-7-1443-2010

[12] Levin LA, Dayton PK (2009) Ecological theory and continental margins: where shallow meets deep. Trends in Ecology and Evolution 24: 606–617. 10.1016/j.tree.2009.04.012 19692143

[13] Grootes PM, Stuiver M (1997) 18O/16O variability in Greenland snow and ice with 10–3 to 105 Yr time resolution. Journal of Geophysical Research 102: 26455–26470. 10.1029/97JC00880

[14] Watson EB, Wasson K, Pasternack GB, Woolfolk A, Van Dyke E, et al (2011) Applications from paleoecology to environmental management and restoration in a dynamic coastal environment. Restoration Ecology 19: 765–775. 10.1111/j.1526-100X.2010.00722.x

[15] Willard DA, Cronin TM (2007) Paleoecology and ecosystem restoration: case studies from Chesapeake Bay and the Florida Everglades. Frontiers in Ecology and the Environment 5: 591–498. 10.1890/070015

[16] van Geen A, Zheng Y, Bernhard JM, Cannariato KG, Carriquiry J, et al (2003) On the preservation of laminations along the western margin of North America. Paleoceanography 18 10.1029/2003PA000911

[17] Cartapanis O, Tachikawa K, Bard E (2011) Northeastern Pacific oxygen minimum zone variability over the past 70 kyr: impact of biological production and oceanic ventilation. Paleoceanography 26 10.1029/2011PA002126

[18] Wyrtki K (1962) The oxygen minima in relation to ocean circulation. Deep-Sea Research 9: 11–23.

[19] Somes CJ, Schmittner A, Galbraith ED, Lehmann MF, Altabet MA, et al (2010) Simulating the global distribution of nitrogen isotopes in the ocean. Global Biogeochemical Cycles 24 10.1029/2009GB003767

[20] Robinson RS, Kienast M, Albuquerque AL, Altabet M, Contreras S, et al (2012) A review of nitrogen isotopic alteration in marine sediments. Paleoceanography 27 10.1029/2012PA002321

[21] Levin LA (2003) Oxygen minimum zone benthos: Adaptation and community response to hypoxia. Oceanography and Marine Biology: An Annual Review 41: 1–45.

[22] Levin LA, Thomas CL, Wishner K (1991) Control of deep-sea benthic community structure by oxygen and organic-matter gradients in the eastern Pacific Ocean. Journal of Marine Research 49: 763–800. 10.1357/002224091784995756

[23] Levin LA, Gage JD, Martin C, Lamont PA (2000) Macrobenthic community structure within and beneath the oxygen minimum zone, NW Arabian Sea. Deep-Sea Research Part II 47: 189–226. 10.1016/S0967-0645(99)00103-4

[24] Hofmann AF, Peltzer ET, Walz PM, Brewer PG (2011) Hypoxia by degrees: Establishing definitions for a changing ocean. Deep-Sea Research Part I 58: 1212–1226. 10.1016/j.dsr.2011.09.004

[25] Emery KO, Hülsemann J (1961) The relationships of sediments, life and water in a marine basin. Deep-Sea Research 8: 3–4.

[26] Keigwin LD, Jones GA (1990) Deglacial climatic oscillations in the Gulf of California. Paleoceanography 5: 1009–1023. 10.1029/PA005i006p01009

[27] Behl RJ, Kennett JP (1996) Brief interstadial events in the Santa Barbara basin, NE Pacific, during the past 60 kyr. Nature 379: 243–246. 10.1038/379243a0

[28] Soutar A, Crill PA (1977) Sedimentation and climatic patterns in the Santa Barbara Basin during the 19^th^ and 20^th^ centuries. Geological Society of America Bulletin 88: 1161–1172. 10.1130/0016-7606(1977)88<1161:SACPIT>2.0.CO;2

[29] Behl RJ (1995) Sedimentary facies and sedimentology of the late quaternary Santa Barbara Basin, Site 893A. The Proceedings of the Ocean Drilling Program Scientific Results 146: 295–308.

[30] Levinton JS (1970) The paleoecological significance of opportunistic species. Lethaia 3: 69–78. 10.1111/j.1502-3931.1970.tb01264.x

[31] Schmiedl G, Mitschele A, Beck S, Emeis KC, Hemleben C, et al (2003) Benthic foraminiferal record of ecosystem variability in the eastern Mediterranean Sea during times of sapropel S5 and S6 deposition. Palaeogeography, Palaeoclimatology, Palaeoecology 190: 139–164. 10.1016/S0031-0182(02)00603-X

[32] Bernhard JM, Reimers CE (1991) Benthic foraminiferal population fluctuations related to anoxia: Santa Barbara Basin. Biogeochemistry 15: 127–149. 10.1007/BF00003221

[33] Corliss BH, Silva KA (1993) Rapid growth of deep-sea benthic foraminifera. Geology 21: 991–994. 10.1130/0091-7613(1993)021<0991:RGODSB>2.3.CO;2

[34] Sen Gupta BK, Turner RE, Rabalais NN (1996) Seasonal oxygen depletion in continental shelf waters of Louisiana-Historical record of benthic foraminifers. Geology 24: 227–230. 10.1130/0091-7613(1996)024<0227:SODICS>2.3.CO;2

[35] Brandes JA, Devol AH, Deutsch C (2007) New developments in the marine nitrogen cycle. Chemical Reviews 107: 577–589. 10.1021/cr050377t 17300141

[36] Francois R (1988) A study on the regulation of the concentrations of some trace metals (Rb, Sr, Zn, Pb, Cu, V, Cr, Ni, Mn, and Mo) in Saanich Inlet sediments, British Columbia, Canada. Marine Geology 83: 285–308. 10.1016/0025-3227(88)90063-1

[37] Altabet MA, Francois R (1994) Sedimentary nitrogen isotopic ratio as a recorder for surface ocean nitrate utilization. Global Biogeochemical Cycles 8: 103–116. 10.1029/93GB03396

[38] Altabet MA, Francois R (1994b) The use of nitrogen isotopic ratio for reconstruction of past changes in surface ocean nutrient utilization. In: Zahn R, Kaminski M, Labeyrie L, Pedersen TF, editors. Carbon Cycling in the Glacial Ocean: Constraints on the Ocean’s Role in Global Change. Heidelberg, New York: Springer pp. 281–306.

[39] Altabet MA, Pilskan C, Thunell R, Pride C, Sigman D, et al (1999) The nitrogen isotope biogeochemistry of sinking particles from the margin of the eastern North Pacific. Deep-Sea Research Part I 46: 655–679. 10.1016/S0967-0637(98)00084-3

[40] Zheng Y, van Geen A, Anderson RF, Gardner JV, Dean WE (2000) Intensification of the northeast Pacific oxygen minimum zone during the Bølling-Allerød warm period. Paleoceanography 15: 528–536. 10.1029/1999PA000473

[41] Nameroff TJ, Balistrier LS, Murray JW (2002) Suboxic trace metal geochemistry in the eastern tropical North Pacific. Geochimica et Cosmochimica Acta 66: 1139–1158. 10.1016/S0016-7037(01)00843-2

[42] Ivanochko TS, Pedersen TF (2004) Determining the influences of Late Quaternary ventilation and productivity variations on Santa Barbara Basin sedimentary oxygenation: a multi-proxy approach. Quaternary Science Reviews 23: 467–480. 10.1016/j.quascirev.2003.06.006

[43] Zheng Y, Anderson RF, van Geen A, Kuwabara J (2000) Authigenic molybdenum formation in marine sediments: a link to porewater sulfide in the Santa Barbara Basin. Geochimica et Cosmochimica Acta 64: 4165–4178. 10.1016/S0016-7037(00)00495-6

[44] del Giorgio PA, Duarte CM (2002) Respiration in the open ocean. Nature 240: 379–384. 10.1038/nature01165 12459775

[45] Broecker WS (1982) Ocean chemistry during glacial time. Geochimica et Cosmochimica Acta 46: 1689–1705. 10.1016/0016-7037(82)90110-7

[46] Tappan H (1986) Phytoplankton: Below the salt at the global table. Journal of Paleontology 60: 545–554.

[47] Curry WB, Crowley TJ (1987) The δ^13^C of equatorial Atlantic surface waters: Implications for ice age pCO_2_ levels. Paleoceanography 2: 489–517. 10.1029/PA002i005p00489

[48] Lambeck K, Smither C, Johnston P (1998) Sealevel change, glacial rebound and mantle viscosity for northern Europe. Geophysical Journal International 134: 102–144. 10.1046/j.1365-246x.1998.00541.x

[49] Peltier WR (1998) Postglacial variation is the level of the sea: Implications for climate dynamics and solid-Earth geophysics. Research in Geophysics 36: 603–689. 10.1029/98RG02638

[50] Becker JJ, Sandwell WH, Smith F, Braud J, Binder B, et al (2009) Global Bathymetry and Elevation Data at 30 Arc Seconds Resolution: SRTM30_PLUS. Marine Geodesy 32: 355–371. 10.1080/01490410903297766

[51] ESRI ESRI (2009) ArcMap 9.2. Redlands, California: ESRI.

[52] Stabeno PJ, Bond NA, Hermann AJ, Kachel NB, Mordy CW, et al (2004) Meteorology and oceanography of the Northern Gulf of Alaska. Continental Shelf Research 24: 859–897. 10.1016/j.csr.2004.02.007

[53] Talley LD (1993) Distribution and formation of North Pacific Intermediate Water. Journal of Physical Oceanography 23: 517–537. 10.1175/1520-0485(1993)023<0517:DAFONP>2.0.CO;2

[54] Lynn RJ, Simpson JJ (1987) The California Current System: The seasonal variability of its physical characteristics. Journal of Geophysical Research 92: 12947–12966. 10.1029/JC092iC12p12947

[55] Hood RR, Abbott MR, Huyer A, Kosro PM (1990) Surface patterns in temperature, flow, phytoplankton biomass, and species composition in the coastal transition zone off northern California. Journal of Geophysical Research 95: 18081–18094. 10.1029/JC095iC10p18081

[56] Paulmier AD, Ruiz-Pino VG (2009) Oxygen minimum zones (OMZs) in the modern ocean. Progress in Oceanography 80: 113–128. 10.1016/j.pocean.2008.08.001

[57] Kaufman D, Manley WF (2004) Pleistocene Maximum and Late Wisconsinan glacier extents across Alaska, USA. In: Ehlers J, Gibbard PL, editors. Quaternary Glaciations: extent and chronology, Part II: North America. Amsterdam: Elsevier pp. 9–27.

[58] Davies MH, Mix AC, Stoner JS, Addison JA, Jaeger J, et al (2011) The deglacial transition on the southeastern Alaska Margin: Meltwater input, sea level rise, marine productivity, and sedimentary anoxia. Paleoceanography 26 10.1029/2010PA002051

[59] Hopkins DM (1959) Cenozoic History of the Bering Land Bridge: The seaway between the Pacific and Arctic basins has often been a land route between Siberia and Alaska. Science 129: 1519–1528. 10.1126/science.129.3362.1519 17742336

[60] Zahn R, Pedersen TF, Bornhold BD, Mix AC (1991) Water mass conversion in the glacial subarctic Pacific (54° N, 148° W): Physical constraints and the benthic-planktonics stable isotope record. Paleoceanography 6: 543–560. 10.1029/91PA01327

[61] Rea DK, Basov IA, Krissek LA (1995) Scientific results of drilling the North Pacific Transect, in North Pacific Transect: Leg 145. The Proceedings of the Ocean Drilling Program Scientific Results 145: 577–596.

[62] McDonald D, Pedersen TF, Crusius J (1999) Multiple late Quaternary episodes of exceptional diatom production in the Gulf of Alaska. Deep-Sea Research Part II 46: 2993–3017. 10.1016/S0967-0645(99)00091-0

[63] Galbraith ED, Jaccard SL, Pedersen TF, Sigman DM, Haug GH, et al (2007) Carbon dioxide release from the North Pacific abyss during the last deglaciation. Nature 449: 890–893. 10.1038/nature06227 17943127

[64] deVernal A, Pedersen TF (1997) Micropaleontology and palynology of core PAR87A-10: A 23,000 year record of paleoenvironmental changes in the Gulf of Alaska, northeast North Pacific. Paleoceanography 12: 821–830. 10.1029/97PA02167

[65] Galbraith ED, Kienast M, Jaccard SL, Pedersen TF, Brunelle BG, et al (2008) Consistent relationship between global climate and surface nitrate utilization in the western subarctic Pacific throughout the last 500 ka. Paleoceanography 23 10.1029/2007PA001518

[66] Broecker WS (1994) Massive iceberg discharges as triggers for global climate change. Nature 372: 421–424. 10.1038/372421a0

[67] Addison JA, Finney BP, Dean WE, Davies MH, Mix AC, et al (2012) Productivity and sedimentary δ^15^N variability for the last 17,000 years along the northern Gulf of Alaska continental slope. Paleoceanography 27 10.1029/2011PA002161

[68] Crusius J, Pedersen TF, Kienast S, Keigwin L, Labeyrie L (2004) Influence of northwest Pacific productivity on North Pacific Intermediate Water oxygen concentrations during the Bølling-Allerød interval (14.7–12.9 ka). Geology 32: 633–636. 10.1130/G20508.1

[69] Duplessey JC, Arnold M, Bard E, Juillet-Leclerc A, Kallel N, et al (1989) AMS C^14^ study of transient events and of the ventilation rate of the Pacific intermediate water during the last deglaciation. Radiocarbon 31: 493–502.

[70] Keigwin LD, Jones GA, Froelich PNA (1992) A 15,000 year paleoenvironmental record from Meiji Seamount, far Northwestern Pacific. Earth and Planetary Science Letters 111: 425–440. 10.1016/0012-821X(92)90194-Z

[71] Keigwin LD (1998) Glacial-age hydrography of the far northwest Pacific Ocean. Paleoceanography 13: 323–339. 10.1029/98PA00874

[72] Barron JA, Bukry D, Dean WE, Addison JA, Finney B (2009) Paleoceanography of the Gulf of Alaska during the past 15,000 years: results from diatoms, silicoflagellates, and geochemistry. Marine Micropaleontology 72: 176–195. 10.1016/j.marmicro.2009.04.006

[73] Jaccard SL, Galbraith ED, Sigman DM, Haug GH, Francois R, et al (2009) Subarctic Pacific evidence for a glacial deepening of the oceanic respired carbon pool. Earth and Planetary Science Letters 277: 156–165. 10.1016/j.epsl.2008.10.017

[74] Jaccard SL, Galbraith ED (2013) Direct ventilation of the North Pacific did not reach the deep ocean during the last deglaciation. Geophysical Research Letters 40: 199–203. 10.1029/2012GL054118

[75] Wyllie JG (1966) Geostrophic flow of the California Current at the surface and at 200 m. CALCOFI Atlas 14. La Jolla, CA: State of Calif. Mar. Re. Comm 12 p.

[76] Hickey BM (1979) The California Current system – hypotheses and facts. Progress in Oceanography 8: 191–279. 10.1016/0079-6611(79)90002-8

[77] Chelton DE (1982) Large-scale response of the California Current to forcing by the wind stress curl. California Cooperative Oceanic Fisheries Investigations 23: 130–148.

[78] Huyer A (1983) Coastal upwelling in the California Current system. Progress in Oceanography 12: 259–284. 10.1016/0079-6611(83)90010-1

[79] Reid JLJ, Schwartzlose RA (1962) Direct measurements of the Davidson Current off central California. Journal of Geophysical Research 67: 2491–2497. 10.1029/JZ067i006p02491

[80] Gardner JV, Hemphill-Haley E (1986) Evidence for a stronger oxygen-minimum zone off central California in Late Pleistocene and Holocene time. Geology 14: 691–694. 10.1130/0091-7613(1986)14<691:EFASOZ>2.0.CO;2

[81] Anderson RY, Hemphill-Haley E, Gardner JV (1987) Persistent late Pleistocene-Holocene seasonal upwelling and varves off the coast of California. Quaternary Research 28: 307–313. 10.1016/0033-5894(87)90069-X

[82] Dean WE, Gardner JV, Anderson RY (1994) Geochemical evidence for enhanced preservation of organic matter in the oxygen minimum zone of the continental margin of northern California during the late Pleistocene. Paleoceanography 9: 47–61. 10.1029/93PA02829

[83] Kennett JP (1995) Latest Quaternary benthic oxygen and carbon isotope stratigraphy: Hole 893A, Santa Barbara Basin, California. The Proceedings of the Ocean Drilling Program Scientific Results 146: 3–18.

[84] Kennett JP, Ingram BL (1995) A 20,000-year record of ocean circulation and climate change from the Santa Barbara basin. Nature 377: 510–513. 10.1038/377510a0

[85] Hendy IL, Kennett JP (1999) Latest Quaternary North Pacific surface-water responses imply atmosphere-driven climate instability. Geology 27: 291–294. 10.1130/0091-7613(1999)027<0291:LQNPSW>2.3.CO;2

[86] Hendy IL, Kennett JP (2000) Dansgaard-Oeschger cycles and the California Current System: Planktonic foraminifera response to rapid climate change in Santa Barbara Basin, Ocean Drilling Program hole 893A. Paleoceanography 15: 30–42. 10.1029/1999PA000413

[87] Hendy IL, Kennett JP (2003) Tropical forcing of North Pacific intermediate water distribution during Late Quaternary rapid climate change? Quaternary Science Reviews 22: 673–689. 10.1016/S0277-3791(02)00186-5

[88] Hendy IL, Kennett JP, Roark EP, Ingram BL (2002) Apparent synchronicity of submillennial scale climate events between Greenland and Santa Barbara Basin, California from 30–10 ka. Quaternary Science Reviews 21: 1167–1184. 10.1016/S0277-3791(01)00138-X

[89] Hill TM, Kennett JP, Pak DK, Behl RJ, Robert C, et al (2006) Pre-Bølling warming in Santa Barbara Basin, California; surface and intermediate water records of early deglacial warmth. Quaternary Science Reviews 25: 2835–2845. 10.1016/j.quascirev.2006.03.012

[90] Hendy IL, Pedersen TF (2006) Oxygen minimum zone expansion in the eastern tropical North Pacific during deglaciation. Geophysical Research Letters 33 10.1029/2006GL025975

[91] Cannariato KG, Kennett JP, Behl RJ (1999) Biotic response to late Quaternary rapid climate switches in Santa Barbara Basin: Ecological and evolutionary implications. Geology 27: 63–66. 10.1130/0091-7613(1999)027<0063:BRTLQR>2.3.CO;2

[92] McKay JL, Pedersen TF, Southon J (2005) Intensification of the oxygen minimum zone in the northeast Pacific off Vancouver Island during the last deglaciation: Ventilation and/or export production? Paleoceanography 20 10.1029/2003PA000979

[93] Cannariato KG, Kennett JP (1999) Climatically related millennial-scale fluctuations in strength of California margin oxygen-minimum zone during the past 60 k.y. Geology 27: 976–978. 10.1130/0091-7613(1999)027<0975:CRMSFI>2.3.CO;2

[94] Hendy IL, Pedersen TF (2005) Is pore water oxygen content decoupled from productivity on the California Margin? Trace element results from Ocean Drilling Program Hole 1017E, San Lucia slope, California. Paleoceanography 20 10.1029/2004PA001123

[95] van Geen A, Fairbanks RG, Dartnell P, McGann M, Gardner JV, et al (1996) Ventilation changes in the northeast Pacific during the last deglaciation. Paleoceanography 11: 519–528. 10.1029/96PA01860

[96] Gardner JV, Dean WE, Dartnell P (1997) Biogenic sedimentation beneath the California Current system for the past 30 ka and its paleoceanographic significance. Paleoceanography 12: 207–225. 10.1029/96PA03567

[97] McGann M (2011) Paleoceanographic changes on the Farallon Escarpment off central California during the last 16,000 years. Quaternary International 235: 26–39. 10.1016/j.quaint.2010.09.005

[98] Stein R, Rack FR (1995) A 160,000-year high-resolution record of quantity and composition of organic carbon in the Santa Barbara Basin (Site 893). In: Kennett JP, Baldauf JG, Lyle M, editors. The Proceedings of the Ocean Drilling Program Scientific Results. College Station, TX: National Science Foundation pp. 125–138.

[99] Gardner JV, Dartnell P (1995) Centennial-scale late Quaternary stratigraphies of carbonate and organic carbon from Santa Barbara Basin, Hole 893A, and their paleoceanographic significance. The Proceedings of the Ocean Drilling Program Scientific Results 146: 103–124.

[100] Emmer E, Thunell RC (2000) Nitrogen isotope variations in Santa Barbara Basin sediments: Implications for denitrification in the eastern tropical North Pacific during the last 50,000 years. Paleoceanography 15: 377–387. 10.1029/1999PA000417

[101] Mix AC, Lund DC, Pisias NP, Bodén P, Bornmalm L, et al (1999) Rapid climate oscillations in the Northeast Pacific during the last deglaciation reflect Northern and Southern hemisphere sources. Geophysical Monograph Series 112: 128–148.

[102] Ohkushi K, Kennett JP, Zeleski CM, Moffitt SE, Hill TM, et al (2013) Quantified intermediate water oxygenation history of the northeast Pacific: A new benthic foraminiferal record from Santa Barbara Basin. Paleoceanography 29: 1–15.

[103] Moffitt SE, Hill TM, Ohkushi K, Kennett JP, Behl RJ (2014) Vertical oxygen minimum zone oscillation since 20 ka in Santa Barbara Basin: A benthic foraminiferal community perspective. Paleoceanography 29 10.1002/2013PA002483

[104] Gardner JV, Dean WE, Kayan R (1992) Paleoceanography of the California Current: Cruise Report, USGS Cruise F2-92, Central and Southern California Margin. US Geological Survey. 285 p.

[105] Dean WE (2007) Sediment geochemical records of productivity and oxygen depletion along the margin of Western North America during the past 60,000 years: teleconnections with Greenland Ice and the Cariaco Basin. Quaternary Science Reviews 26: 89–114. 10.1016/j.quascirev.2006.08.006

[106] Cuffey KM, Clow GD (1997) Temperature, accumulation, and ice sheet elevation in central Greenland through the last deglacial transition. Journal of Geophysical Research 102: 26383–26396. 10.1029/96JC03981

[107] Alley RB (2000) The Younger Dryas cold interval as viewed from central Greenland. Quaternary Science Reviews 19: 213–226. 10.1016/S0277-3791(99)00062-1

[108] Fiedler PC, Talley LD (2006) Hydrography of the eastern tropical Pacific: A review. Progress in Oceanography 69: 143–180. 10.1016/j.pocean.2006.03.008

[109] Wang C, Enfield DB (2001) The tropical Western Hemisphere warm pool. Geophysical Research Letters 28: 1635–1638. 10.1029/2000GL011763

[110] Reid JL (1965) Intermediate waters of the Pacific Ocean La Jolla, CA: Scripps Institution of Oceanography.

[111] Hickey BM (1998) Coastal oceanography of western North America from the tip of Baja California to Vancouver Island. In: Robinson AR, Brink KH, editors. The Sea: The Global Coastal Ocean New York: Wiley pp. 345–393.

[112] Fine RA, Maillet KA, Sullivan KF, Willey D (2001) Circulation and ventilation flux of the Pacific Ocean. Journal of Geophysical Research 106: 22159–22178. 10.1029/1999JC000184

[113] Bostock HC, Opdyke BN, Williams MJM (2010) Characterizing the intermediate depth waters of the Pacific Ocean using δ^13^C and other geochemical tracers. Deep-Sea Research Part I 57: 847–859. 10.1016/j.dsr.2010.04.005

[114] Wyrtki K (1966) Oceanography of the eastern equatorial Pacific Ocean. Oceanography and Marine Biology: An Annual Review 4: 33–68.

[115] O’Connor BM, Fine RA, Maillet KA, Olson DB (2002) Formation rates of subtropical underwater in the Pacific Ocean. Deep-Sea Research Part I 49: 1571–1590. 10.1016/S0967-0637(02)00087-0

[116] Montgomery RB, Stroup ED (1962) Equatorial waters and currents at 150° W in July–August 1952. Johns Hopkins Oceanographic Study 1: 68.

[117] Tsuchiya M (1981) The origin of the Pacific equatorial 13°C Journal of Physical Oceanography 11: 794–812. 10.1175/1520-0485(1981)011<0794:TOOTPE>2.0.CO;2

[118] Hanawa K, Talley LD (2001) Mode waters. In: Siedler G, Church J, Gould J, editors. Ocean Circulation and Climate: Observing and Modeling the Global Ocean. New York: Academic Press pp. 373–386.

[119] Nameroff TJ, Calvert SE, Murray JW (2004) Glacial-interglacial variability in the eastern tropical North Pacific oxygen minimum zone recorded by redox sensitive trace metals. Paleoceanography 19 10.1029/2003PA000912

[120] Ganeshram RS, Pedersen TF (1998) Glacial interglacial variability in upwelling and bioproductivity off NW Mexico: Implication to Quaternary Paleoclimate. Paleoceanography 13: 634–645. 10.1029/98PA02508

[121] Ortiz JD, O’Connell SB, DelViscio J, Dean W, Carriquiry JD, et al (2004) Enhanced marine productivity off western North America during warm climate intervals of the past 52 k.y. Geology 32: 521–524. 10.1130/G20234.1

[122] Kienast SS, Calvert SE, Pedersen TF (2002) Nitrogen isotope and productivity variations along the northeast Pacific margin over the last 120 kyr: Surface and subsurface paleoceanography. Paleoceanography 17 10.1029/2001PA000650

[123] De Pol-Holz R, Ulloa O, Dezileau L, Kaiser J, Lamy F, et al (2006) Melting of the Patagonian Ice Sheet and deglacial perturbations of the nitrogen cycle in the eastern South Pacific. Geophysical Research Letters 33 10.1029/2005GL024477

[124] Robinson RS, Martinez P, Pena LD, Cacho I (2009) Nitrogen isotopic evidence for deglacial changes in nutrient supply in the eastern equatorial Pacific. Paleoceanography 24 10.1029/2008PA001702

[125] Pichevin LE, Ganeshram RS, Francavilla S, Arellano-Torres E, Pedersen TF, et al (2010) Interhemispheric leakage of isotopically heavy nitrate in the eastern tropical Pacific during the last glacial period. Paleoceanography 25 10.1029/2009PA001754

[126] Ganeshram RS, Pedersen TF, Calvert SE, Murray JW (1995) Large changes in ocean nutrient inventories from glacial to interglacial periods. Nature 376: 755–758. 10.1038/376755a0

[127] Ganeshram RS, Pedersen TF, Calvert SE, McNeill GW, Fontugne MR (2000) Glacial-interglacial variability in denitrification in the world’s ocean: Causes and consequences. Paleoceanography 15: 361–376. 10.1029/1999PA000422

[128] Pride C, Thunell RC, Sigman D, Keigwin L, Altabet M, et al (1999) Nitrogen isotopic variations in the Gulf of California since the last deglaciation Response to global climate change. Paleoceanography 14: 397–409. 10.1029/1999PA900004

[129] Blanchet CL, Thouveny N, Vidal L, Leduc G, Tachikawa K, et al (2007) Terrigenous input response to glacial/interglacial climatic variations over southern Baja California: A rock magnetic approach. Quaternary Science Reviews 26: 3118–3133. 10.1016/j.quascirev.2007.07.008

[130] Daneri G, Dellarossa V, Quiñones R, Jacob B, Montero P, et al (2000) Primary production and community respiration in the Humboldt Current System off Chile and associated oceanic regions. Marine Ecology Progress Series 197: 41–49. 10.3354/meps197041

[131] Sellanes J, Neira C, Quiroga E, Teixido N (2010) Diversity patterns along and across the Chilean margin: a continental slope encompassing oxygen gradients and methane seep benthic habitats. Marine Ecology 31: 111–124. 10.1111/j.1439-0485.2009.00332.x

[132] Silva N, Rojas N, Fedele A (2009) Water masses in the Humboldt Current system: properties, distribution, and the nitrate deficit as a chemical water mass tracer for equatorial subsurface water off Chile. Deep-Sea Research Part II 56: 1004–1020. 10.1016/j.dsr2.2008.12.013

[133] Silva SN, Neshyba S (1979) On the southernmost extension of the Peru-Chile Undercurrent. Deep-Sea Research 26: 1387–1393. 10.1016/0198-0149(79)90006-2

[134] Schneider W, Fuenzalida R, Rodriguez-Rubio E, Garcs-Vargas J (2003) Characteristics and formation of Eastern South Pacific Intermediate Water. Geophysical Research Letters 30 10.1029/2003GL017086

[135] Fuenzalida R, Schneider W, Garcés-Vargas J, Bravo L, Lange C (2009) Vertical and horizontal extension of the oxygen minimum zone in the eastern South Pacific Ocean. Deep-Sea Research Part II 56: 992–1003. 10.1016/j.dsr2.2008.11.001

[136] Molina V, Farías L, Eissler Y, Cuevas LA, Morales CE, et al (2005) Ammonium cycling under a strong oxygen gradient associated with the oxygen minimum zone off northern Chile (~23° S). Marine Ecology Progress Series 288: 35–43. 10.3354/meps288035

[137] Farías L, Paulmier A, Gallegos M (2007) Nitrous oxide and N-nutrient cycling in the oxygen minimum zone off northern Chile. Deep-Sea Research Part I 54: 164–180. 10.1016/j.dsr.2006.11.003

[138] Toggweiler J, Dixon K, Broecker W (1991) The Peru upwelling and the ventilation of the South Pacific thermocline. Journal of Geophysical Research 96: 20467–20497. 10.1029/91JC02063

[139] Toggweiler JR, Carson S (1995) What are upwelling systems contributing to the ocean’s carbon and nutrient budgets? In: Summerhayes CP, Emeis KC, Angel MV, Smith RL, Zeitzschel B et al, editors. Upwelling in the Ocean, Modern Processes and Ancient Records. Hoboken, New Jersey: John Wiley pp. 337–361.

[140] Dugdale RC, Wischmeyer AG, Wilkerson FP, Barber RT, Chai F, et al (2002) Meridional asymmetry of source nutrients to the equatorial Pacific upwelling ecosystem and its potential impact on ocean-atmospheric CO_2_ flux: A data and modeling approach. Deep-Sea Research Part II 49: 2513–2531. 10.1016/S0967-0645(02)00046-2

[141] Mackas D, Strub PT, Thomas A, Montecino V (2006) Eastern ocean boundaries pan-regional overview. In: Robinson AR, Brink KH, editors. The Sea. Cambridge: Harvard Univ. Press pp. 21–59.

[142] Reid JL (1997) On the total geostrophic circulation of the Pacific Ocean: flow patterns, tracers, and transports. Progress in Oceanography 39: 263–352. 10.1016/S0079-6611(97)00012-8

[143] Wijffels SE, Toole JM, Bryden HL, Fine RA, Jenkins WJ, et al (1996) The water masses and circulation at 10° N in the Pacific. Deep-Sea Research 43: 501–544. 10.1016/0967-0637(96)00006-4

[144] Bradtmiller LI, Anderson RF, Sachs JP, Fleisher MQ (2010) A deeper respired carbon pool in the glacial equatorial Pacific Ocean. Earth and Planetary Science Letters 299: 417–425. 10.1016/j.epsl.2010.09.022

[145] de Vries TJ, Schrader H (1981) Variation of upwelling/oceanic conditions during the latest Pleistocene through Holocene off the Peruvian coast: a diatom record. Marine Micropalentology 6: 157–167. 10.1016/0377-8398(81)90003-7

[146] Petit JR, Jouzel J, Raynaud D, Barkov NI, Barnola JM, et al (1999) Climate and Atmospheric History of the Past 420,000 years from the Vostok Ice Core, Antarctica. Nature 399: 429–436. 10.1038/20859

[147] Petit JR, Jouzel J, Raynaud D, Barkov NI, Barnola JM, et al (2001) Vostok ice core data for 420,000 years. IGBP PAGES/World Data Center for Paleoclimatology Data Contribution Series 76.

[148] Yang YL, Elderfield H, Pedersen TF, Ivanovich M (1995) Geochemical record of the Panama basin during the last glacial maximum carbon event shows that the glacial ocean was not anoxic. Geology 23: 1115–1118. 10.1130/0091-7613(1995)023<1115:GROTPB>2.3.CO;2

[149] Kienast SS, Kienast M, Mix AC, Calvert SE, Francois R (2007) Thorium-230 normalized particle flux and sediment focusing in the Panama Basin region during the last 30,000 years. Paleoceanography 22 10.1029/2006PA001357

[150] Mohtadi M, Hebbeln D (2004) Mechanisms and variations of the paleoproductivity off northern Chile (24° S–33° S) during the last 40,000 years. Paleoceanography 19 10.1029/2004PA001003

[151] Higginson MJ, Altabet MA (2004) Initial test of the silicic acid leakage hypothesis using sedimentary biomarkers. Geophysical Research Letters 31 10.1029/2004GL020511

[152] Andrews WRH, Hutchings L (1980) Upwelling in the southern Benguela Current. Progress in Oceanography 9: 1–81. 10.1016/0079-6611(80)90015-4

[153] Calvert SE, Price NB (1970) Minor metal contents of recent organic-rich sediments off south west Africa. Nature 227: 593–595. 10.1038/227593a0 16058063

[154] Bailey GW (1991) Organic carbon flux and development of oxygen deficiency on the modern Benguela continental shelf south of 22° S: Spatial and temporal variability. The Geological Society Special Publications 58: 171–183. 10.1144/GSL.SP.1991.058.01.12

[155] Zettler ML, Bochert R, Pollehne F (2009) Macrozoobenthos diversity in an oxygen minimum zone off northern Namibia. Marine Biology 9: 1946–1961.

[156] Diester-Haass L, Meyers PA, Rothe P (1986) Light-dark cycles in opal-rich sediments near the Plio-Pleistocene boundary, DSDP Site 532, Walvis Ridge Continental Terrace. Marine Geology 73: 1–23. 10.1016/0025-3227(86)90108-8

[157] Martinez P, Bertrand P, Shimmield GB, Cochrane K, Jorissen FJ, et al (1999) Upwelling intensity and ocean productivity charges off Cape Blanc (northwest Africa) during the last 70,000 years: geochemical and micropaleontological evidence. Marine Geology 158: 57–74. 10.1016/S0025-3227(98)00161-3

[158] Yu EF, Liang CH, Chen MT (1999) Authigenic uranium in marine sediments of the Benguela Current upwelling region during the last glacial period. Terrestrial, Atmospheric, and Oceanic Sciences 10: 201–214.

[159] Abrantes F (2000) 200,000 yr diatom records from the Atlantic upwelling site reveal maximum productivity during LGM and a shift in phytophlankton community structure at 185,000 yr. Earth Planetary Science Letters 176: 7–16. 10.1016/S0012-821X(99)00312-X

[160] Mollenhauer G, Schneider RR, Müller PJ, Speiβ V, Wefer G (2002) Glacial/interglacial variability in the Benguela upwelling system: Spatial distribution and budgets of organic carbon accumulation. Global Biogeochemical Cycles 16 10.1029/2001GB001488

[161] Gage JD, Levin LA, Wolff GA (2000) Benthic processes in the deep Arabian Sea: introduction and overview. Deep-Sea Research Part II 47: 1–8.

[162] Cowie GL (2005) The biogeochemistry of Arabian Sea surficial sediments: a review of recent studies. Progress in Oceanography 65: 260–289. 10.1016/j.pocean.2005.03.003

[163] Altabet MA, Francois R, Murray DW, Prell WL (1995) Climate-related variations in denitrification in the Arabian Sea from sediment ^15^N/^14^N ratios. Nature 373: 506–509. 10.1038/373506a0

[164] Banse K (1984) Overview of the hydrography and associated biological phenomena in the Arabian Sea, off Pakistan. In: Haq BU, Milliman JD, Nostrand V, editors. Marine Geology and Oceanography of the Arabian Sea and Coastal Pakistan. New York: Van Nostrand Reinhold/​Scientific and Academic Editions pp. 271–303.

[165] Hermelin JOR (1992) Variations in the benthic foraminiferal fauna of the Arabian Sea: a response to changes in upwelling intensity? The Geological Society Special Publications 64: 151–166. 10.1144/GSL.SP.1992.064.01.10

[166] Parulekar AH, Harkantra SN, Ansari ZA, Matondkar SGP (1982) Abyssal benthos of the central Indian Ocean. Deep-Sea Research 29: 1531–1537. 10.1016/0198-0149(82)90041-3

[167] Schulz H, von Rad U, Erlenkeuser H (1998) Correlation between Arabian Sea and Greenland climate oscillations of the past 110,000 years. Nature 393: 54–57.

[168] Reichart GJ, Schenau SJ, de Lange GJ, Zachariasse WJ (2002) Synchroneity of oxygen minimum zone intensity on the Oman and Pakistan Margins at sub-Milankovitch time scales. Marine Geology 185: 403–415. 10.1016/S0025-3227(02)00184-6

[169] Sarkar A, Ramesh R, Bhattacharya SK, Rajagopalan G (1990) Oxygen isotope evidence for a stronger winter monsoon current during the last glaciation. Nature 343: 549–551. 10.1038/343549a0

[170] Sarkar A, Bhattacharya SK, Sarin MM (1993) Geochemical evidence for anoxic deep-water in the Arabian Sea during the last glaciation. Geochimica et Cosmochimica Acta 57: 1009–1016. 10.1016/0016-7037(93)90036-V

[171] von Rad U, Schulz H, Party SS (1995) Sampling the oxygen minimum zone off Pakistan–glacial interglacial variations of anoxia and productivity (preliminary results, Sonne-90 cruise). Marine Geology 125: 7–19. 10.1016/0025-3227(95)00051-Y

[172] Reichart GJ, den Dulk M, Visser HJ, van der Weijden CH, Zachariasse WJ (1997) A 225 kyr record of dust supply, paleoproductivity and the oxygen minimum zone from the Murray Ridge (northern Arabian Sea). Palaeogeography, Palaeoclimatology, Palaeoecology 134: 149–169. 10.1016/S0031-0182(97)00071-0

[173] Altabet MA, Higginson MJ, Murray DW (2002) The effect of millennial-scale changes in Arabian Sea denitrification on atmospheric CO_2_ . Nature 415: 159–162. 10.1038/415159a 11805831

[174] Schulte S, Rostek F, Bard E, Rullkotter J, Marchal O (1999) Variations of oxygen-minimum and primary productivity recorded in sediments of the Arabian Sea. Earth and Planetary Science Letters 173: 205–221. 10.1016/S0012-821X(99)00232-0

[175] Bryan SP, Marchitto TM, Lehman SJ (2010) The release of C^14^-depleted carbon from the deep ocean during the last deglaciation: Evidence from the Arabian Sea. Earth and Planetary Science Letters 298: 244–254. 10.1016/j.epsl.2010.08.025

[176] Reichart GJ, Lourens LJ, Zachariasse WJ (1998) Temporal variability in the northern Arabian Sea Oxygen Minimum Zone (OMZ) during the last 225,000 years. Paleoceanography 13: 607–621. 10.1029/98PA02203

[177] Overpeck J, Anderson D, Trumbore S, Prell W (1996) The southwest Indian Monsoon over the last 18000 years. Climate Dynamics 12: 213–225. 10.1007/BF00211619

[178] Pichevin L, Bard E, Martinez P, Billy I (2007) Evidence of ventilation changes in the Arabian Sea during the late Quaternary: Implication for denitrification and nitrous oxide emission. Global Biogeochemical Cycles 21 10.1029/2006GB002852

[179] Duplessy JC, Arnold M, Bard E, Juillet-Leclerc A, Kallel N, et al (1989) AMS C^14^ study of transient events and of the ventilation rate of the Pacific intermediate water during the last deglaciation. Radiocarbon 31: 493–502.

[180] Hendy IL, Pedersen TF, Kennett JP, Tada R (2004) Intermittent existence of a southern Californian upwelling cell during submillennial climate change of the last 60 kyr. Paleoceanography 9.

[181] Martinez P, Robinson RS (2010) Increase in water column denitrification during the last deglaciation: the influence of oxygen demand in the eastern equatorial Pacific. Biogeosciences 7: 1–9. 10.5194/bg-7-1-2010

[182] Cheshire H, Thurow J, Nederbragt A (2005) Later Quaternary climate change record from two long sediment cores from Guaymas Basin, Gulf of California. Journal of Quaternary Science 20: 457–469. 10.1002/jqs.944

[183] Cartapanis O, Tachikawa K, Bard E (2012) Latitudinal variations in intermediate depth ventilation and biological productivity over northeastern Pacific Oxygen Minimum Zones during the last 60 ka. Quaternary Science Reviews 53: 24–38. 10.1016/j.quascirev.2012.08.009

[184] Deutsch C, Brix H, Ito T, Frenzel H, Thompson L (2011) Climate-forced Variability of ocean hypoxia. Science 333: 336–339. 10.1126/science.1202422 21659566

[185] Bograd SJ, Castro CG, Di Lorenzo E, Palacios DM, Bailey H, et al (2008) Oxygen declines and the shoaling of the hypoxic boundary in the California Current. Geophysical Research Letters 35 10.1029/2008GL034185

[186] Stramma L, Johnson GC, Sprintal J, Mohrholz V (2008) Expanding Oxygen-Minimum Zones in the tropical oceans. Science 320: 655–658. 10.1126/science.1153847 18451300

[187] Stramma L, Schmidtko S, Levin LA, Johnson GC (2010) Ocean oxygen minima expansions and their biological impacts. Deep-Sea Research Part I 57: 587–595. 10.1016/j.dsr.2010.01.005

[188] McClatchie S, Goericke R, Cosgrove R, Auad G, Vetter R (2010) Oxygen in the Southern California Bight: Multidecadal trends and implications for demersal fisheries. Geophysical Research Letters 37 10.1029/2010GL044497

[189] Ono T, Midorikawa T, Watanabe YW, Tadokoro K, Saino T (2001) Temporal increases of phosphate and apparent oxygen utilization in the subsurface waters of western subarctic Pacific from 1968 to 1998. Geophysical Research Letters 28: 3285–3288. 10.1029/2001GL012948

[190] Whitney FA, Freeland HJ, Robert M (2007) Persistently declining oxygen levels in the interior waters of the eastern subarctic Pacific. Progress in Oceanography 75: 179–199. 10.1016/j.pocean.2007.08.007

[191] Emerson S, Mecking S, Abell J (2001) The biological pump in the subtropical North Pacific Ocean: Nutrient sources, Redfield ratios, and recent changes. Global Biogeochemical Cycles 15: 535–554. 10.1029/2000GB001320

[192] Garcia HE, Locarnini RA, Boyer TP, Antonov JI, Baranova OK, et al (2010) Dissolved Oxygen, Apparent Oxygen Utilization, and Oxygen Saturation. In: Levitus S, editor. World Ocean Atlas 2009. Washington, D.C.: U.S. Government Printing Office pp. 344.

[193] Bindoff NL, McDougall TJ (2000) Decadal changes along an Indian ocean section at 32° S and their interpretation. Journal of Physical Oceanography 30: 1207–1222. 10.1175/1520-0485(2000)030<1207:DCAAIO>2.0.CO;2

[194] Matear RJ, Hirst AC (2003) Long-term changes in dissolved oxygen concentrations in the ocean caused by protracted global warming. Global Biogeochemical Cycles 17 10.1029/2002GB001997

[195] Frieder CA, Nam SH, Martz TR, Levin LA (2012) High temporal and spatial variability of dissolved oxygen and pH in a nearshore California kelp forest. Biogeosciences 9: 3917–3930. 10.5194/bg-9-3917-2012

[196] Send U, Nam S (2012) Relaxation from upwelling: The effect on dissolved oxygen on the continental shelf. Journal of Geophysical Research 117 10.1029/2011JC007517

[197] Nam S, Kim HJ, Send U (2011) Amplification of hypoxic and acidic events by La Nina conditions on the continental shelf off California. Geophysical Research Letters 38 10.1029/2011GL049549

[198] Deutsch C, Berelson W, Thunell R, Weber T, Tems C, et al (2014) Centennial changes in North Pacific anoxia linked to tropical trade winds. Science 345: 665–668. 10.1126/science.1252332 25104384

[199] Murray JW (2001) The niche of benthic foraminifera, critical thresholds and proxies. Marine Micropaleontology 41: 1–7. 10.1016/S0377-8398(00)00057-8

[200] Bernhard JM (1986) Characteristic assemblages and morphologies of benthic foraminifera from anoxic, organic-rich deposits: Jurassic through Holocene. Journal of Foraminiferal Research 16: 207–215. 10.2113/gsjfr.16.3.207

[201] Corliss BH (1991) Morphology and microhabitat preferences of benthic foraminifera from the northwest Atlantic Ocean. Marine Micropaleontology 17: 195–236. 10.1016/0377-8398(91)90014-W

[202] Bernhard JM (1992) Benthic foraminiferal distribution and biomass related to pore water oxygen: Central California Continental slope and rise. Deep-Sea Research 39: 586–605. 10.1016/0198-0149(92)90090-G

[203] Jorissen FJ, Fontanier C, Thomas E (2007) Paleoceanographical proxies based on deep-sea benthic foraminiferal assemblage characteristics. In: Hillaire-Marcel C, de Vernal A, editors. Proxies in Late Cenozoic Paleoceanography: Pt 2: Biological tracers and biomarkers. Amsterdam: Elsevier pp. 263–326.

[204] Fontanier C, Jorissen FJ, Licari L, Alexandre A, Anschutz P, et al (2002) Live benthic foraminiferal faunas from the Bay of Biscay: Faunal density, composition, and microhabitats. Deep-Sea Research Part I 49: 751–785. 10.1016/S0967-0637(01)00078-4

[205] Fontanier C, Jorissen FJ, Chaillou G, Anschutz P, Grémare A, et al (2005) Live foraminiferal faunas from a 2800 m deep lower canyon station from the Bay of Biscay: Faunal response to focusing of refractory organic matter. Deep-Sea Research Part I 52: 1189–1227. 10.1016/j.dsr.2005.01.006

[206] Naidu PD, Malmgren BA (1995) Do benthic foraminifer records represent a productivity index in oxygen minimum zone areas? An evaluation from the Oman Margin, Arabian Sea. Marine Micropaleontology 26: 49–55. 10.1016/0377-8398(95)00014-3

[207] Bernhard JM, Buck KR, Farmer MA, Bowser SS (2000) The Santa Barbara Basin is a symbiosis oasis. Nature 403: 77–80. 10.1038/47476 10638755

[208] van Geen A, McCorkle DC, Klinkhammer GP (1995) Sensitivity of the phosphate-cadmium-carbon isotope relation in the ocean to cadmium removal by suboxic sediments. Paleoceanography 10: 159–169. 10.1029/94PA03352

[209] Rosenthal Y, Lam P, Boyle EA, Thomson J (1995) Authigenic cadmium enrichments in suboxic sediments: Precipitation and postdepositional mobility. Earth and Planetary Science Letters 132: 99–111. 10.1016/0012-821X(95)00056-I

[210] Crusius J, Calvert S, Pedersen T, Sage D (1996) Rhenium and molybdenum in sediments as indicators of oxic, suboxic and sulfidic conditions of deposition. Earth and Planetary Science Letters 145: 65–78. 10.1016/S0012-821X(96)00204-X

[211] Klinkhammer GP, Palmer MR (1991) Uranium in the oceans: where it goes and why. Geochimica et Cosmochimica Acta 55: 1799–1806. 10.1016/0016-7037(91)90024-Y

[212] Tribovillard N, Algeo TJ, Lyons T, Riboulleau A (2006) Trace metals as paleoredox and paleoproductivity proxies: An update. Chemical Geology 232: 12–32. 10.1016/j.chemgeo.2006.02.012

[213] Bull D, Kemp AES, Weedon GP (2000) A 160-k.y.-old record of El Niño-Southern Oscillation in marine production and coastal runoff from Santa Barbara Basin, California, USA. Geology 28: 1007–1010. 10.1130/0091-7613(2000)28<1007:AKROEN>2.0.CO;2

[214] Kennett JP, Roark EB, Cannariato KG, Ingram BL, Tada R (2000) Latest Quaternary paleoclimatic and radiocarbon chronology, Hole 1017E, Southern California margin. The Proceedings of the Ocean Drilling Program Scientific Results 167: 249–254.

[215] Barron JA, Heusser L, Herbert T, Lyle M (2003) High resolution climatic evolution of coastal northern California during the past 16,000 years. Paleoceanography 18 10.1029/2002PA000768

[216] Barron JA, Bukry D (2007) Development of the California Current during the past 12,000 yr based on diatoms and silicoflagellates. Palaeogeography, Palaeoclimatology, Palaeoecology 248: 313–338. 10.1016/j.palaeo.2006.12.009

[217] Kienast SS, McKay JL (2001) Sea surface temperatures in the subarctic northeast Pacific reflect millennial-scale climate oscillations during the last 16 kyrs. Geophysical Research Letters 28: 1563–1566. 10.1029/2000GL012543

[218] Keigwin LD (2002) Late Pleistocene-Holocene paleoceanography and ventilation of the Gulf of California. Journal of Oceanography 58: 421–432. 10.1023/A:1015830313175

[219] van Geen A, Melville SPRV (2001) Baja California coring cruise OXMZ01MV: Core descriptions and CTD/Rosette data. Palisades, N. Y.: Lamont-Doherty Earth Obs., Columbia Univ.

[220] Dean WE, Zheng Y, Ortiz JD, van Geen A (2006) Sediment Cd and Mo accumulation in the oxygen minimum zone off western Baja California linked to global climate over the past 52 kyr. Paleoceanography 21 10.1029/2005PA001239

[221] Ganeshram RS, Calvert SE, Pedersen TF, Cowie GL (1999) Factors controlling the burial of organic carbon in laminated and bioturbated sediments off NW Mexico: Implications for hydrocarbon preservation. Geochimica et Cosmochimica Acta 63: 1723–1724. 10.1016/S0016-7037(99)00073-3

[222] Ganeshram RS, Pedersen TF, Calvert SE, François R (2002) Reduced nitrogen fixation in the glacial ocean inferred from changes in marine nitrogen and phosphorus inventories. Nature 415: 156–159. 10.1038/415156a 11805830

[223] Benway HM, Mix AC, Haley BA, Klinkhammer GP (2006) Eastern Pacific Warm Pool paleosalinity and climate variability: 0–30 kyr. Paleoceanography 21 10.1029/2005PA001208

[224] Reimers CE, Suess E (1983) The partitioning of organic carbon fluxes and sedimentary organic matter decomposition rates in the ocean. Marine Chemistry 13: 141–168. 10.1016/0304-4203(83)90022-1

[225] Agnihotri R, Altabet MA, Herbert TD (2006) Influence of marine denitrification on atmospheric N_2_O variability during the Holocene. Geophysical Research Letters 33 10.1029/2006GL025864

[226] Agnihotri R, Altabet MA, Herbert TD, Tierney JE (2008) Subdecadally resolved paleoceanography of the Peru Margin during the last two millennia. Geochemistry Geophysics Geosystems 9 10.1029/2007GC001744

[227] Dubois N, Kienast M, Kienast SS, Normandeau C, Calvert SE, et al (2011) Millennial‐scale variations in hydrography and biogeochemistry in the eastern equatorial Pacific over the last 100 kyr. Quaternary Science Reviews 30: 210–223. 10.1016/j.quascirev.2010.10.012

[228] Dubois N, Kienast M (2011) Spatial reorganization in the equatorial divergence in the Eastern Tropical Pacific during the last 150 kyr. Geophysical Research Letters 38 10.1029/2011GL048325

[229] Kusch S, Eglinton TI, Mix AC, Mollenhauer G (2010) Timescales of lateral sediment transport in the Panama Basin as revealed by radiocarbon ages of alkenones, total organic carbon and foraminifera. Earth and Planetary Science Letters 290: 340–350. 10.1016/j.epsl.2009.12.030

[230] Loubere P, Mekik F, Francois R, Pichat S (2004) Export fluxes of calcite in the eastern equatorial Pacific from the Last Glacial Maximum to present. Paleoceanography 19.

[231] Pedersen TF, Pickering M, Vogel JS, Southon JN, Nelson DE (1988) The response of benthic foraminifera to productivity cycles in the eastern equatorial Pacific: Faunal and geochemical constraints on glacial bottom water oxygen levels. Paleoceanography 3: 157–168. 10.1029/PA003i002p00157

[232] Lea DW, Pak DK, Spero HJ (2000) Climate impact of late Quaternary equatorial Pacific sea surface temperatures variations. Science 289: 1719–1723. 10.1126/science.289.5485.1719 10976060

[233] Martin PA, Lea DW, Rosenthal Y, Shackleton NJ, Sarnthein M, et al (2002) Quaternary deep sea temperatures derived from benthic foraminiferal Mg/Ca. Earth and Planetary Science Letters 198: 193– 209. 10.1016/S0012-821X(02)00472-7

[234] Skinner LC, Shackleton NJ (2005) An Atlantic lead over Pacific deep-water change across Termination I: Implications for the application of the marine isotope stage stratigraphy. Quaternary Science Reviews 24: 571–580. 10.1016/j.quascirev.2004.11.008

[235] Spero HJ, Lea DW (2002) The cause of carbon isotope minimum events at glacial terminations. Science 296: 522–525. 10.1126/science.1069401 11964477

[236] De Pol-Holz R, Ulloa O, Lamy F, Dezileau L, Sabatier P, et al (2007) Late Quaternary variability of sedimentary nitrogen isotopes in the eastern South Pacific. Paleoceanography 22 10.1029/2006PA001308

[237] Shackleton NJ, Imbrie J, Hall MA (1983) Oxygen and carbon isotope record Of East Pacific Core V19–30—implications for the formation of deep-water in the Late Pleistocene North-Atlantic. Earth and Planetary Science Letters 65: 233–244. 10.1016/0012-821X(83)90162-0

[238] Koutavas A, Lynch-Steiglitz J (2003) Glacial-interglacial dynamics of the eastern equatorial Pacific cold tongue-Intertropical Convergence Zone system reconstructed from oxygen isotope records. Paleoceanography 18 10.1029/2003PA000894

